# The recoverability, reliability, and generalizability of reward processing parameters and relation to mental health symptoms

**DOI:** 10.1017/S0033291726105340

**Published:** 2026-07-28

**Authors:** Alexandra C. Pike, Rowan Board, Eoin Travers, Megan Croal, Yeuk Ching Lam, Vincent Valton, Oliver J. Robinson, Jonathan P. Roiser

**Affiliations:** 1 University of York, UK; 2 University College London, UK

**Keywords:** computational modeling, computational psychiatry, depression, measurement, reliability, reward

## Abstract

**Background:**

Theory-driven computational psychiatry attempts to use cognitive models of computation to understand how mental health problems might relate to cognitive processes such as learning and decision-making. However, the potential applications and relevance of this approach are contingent on several (often implicit) assumptions, including that computational parameters 1) are recoverable, 2) are reliable over time, 3) reflect conceptually similar processes across different tasks, and 4) relate to symptoms.

**Methods:**

To illustrate and test these assumptions for a selection of commonly used cognitive tasks, we recruited a large online sample of participants (*n* = 548), who completed seven mental health questionnaires and five tasks. A subset of *n* = 115 re-completed the tasks 14 days later. For each task, five models were fit, and the winning model was selected through Bayesian model comparison.

**Results:**

The parameters of the winning models showed excellent recovery and good-to-excellent test-retest reliability. Parameters that were theoretically similar to each other were, however, generally not related, with relationships only found within the class of ‘inverse temperature’-like parameters. Finally, only parameters from two of the five tasks (four-armed bandit and cognitive effort) related to symptoms, and these relationships were weak.

**Conclusions:**

It seems that at least some of the implicit assumptions made when advocating for the use of computational models in psychiatry are not met for these popular tasks and computational models. Computational psychiatry researchers should carefully assess the assumptions and psychometric properties of their tasks and models as part of their early-phase development to ensure robustness of the field going forward.

## Introduction

One of the key goals of computational psychiatry is to understand how mental health problems relate to (or are caused by) changes in cognitive processes such as learning and decision-making (Adams, Huys, & Roiser, 2016; Friston, Stephan, Montague, & Dolan, 2014; Huys, Maia, & Frank, 2016; Kishida, King-Casas, & Montague, 2010; Maia & Frank, 2011; Montague, Dolan, Friston, & Dayan, 2012; Paulus, Huys, & Maia, 2016). Computational psychiatry researchers often propose that identifying such cognitive changes might improve personalized approaches to treatment, mechanistic understanding of the development and maintenance of illness, and identification of biomarkers for clinical trials (Browning et al., 2020). However, this promise rests on a number of assumptions – which are increasingly coming under scrutiny (Brown, Chen, Gillan, & Price, 2020; Eckstein, Wilbrecht, & Collins, 2021; Eckstein et al., 2022; Haines et al., 2025; Karvelis, Paulus, & Diaconescu, 2023; Katahira, Oba, & Toyama, 2024; Mkrtchian, Valton, & Roiser, 2023; Palminteri, Wyart, & Koechlin, 2017; Pike et al., 2024; Schaaf, Weidinger, Molleman, & van den Bos, 2024; Schurr et al., 2024; Toyama, Katahira, & Kunisato, 2023; Vrizzi et al., 2025; Wilson & Collins, 2019). In this paper, we enumerate these assumptions (for a useful schematic, see Karvelis, Paulus, & Diaconescu, 2023), and present data from a test case that illustrates them further.

The first assumption of the computational psychiatry approach is that model parameters can be recovered reliably – i.e. that the estimated parameters are similar when model-fitting is repeated; or, relatedly, model-fitting of synthetic data generated by known parameters outputs the original generating parameter values (Karvelis, Paulus, & Diaconescu, 2023; Wilson & Collins, 2019). If not, then this puts an upper bound on the strength of associations between parameters and other relevant variables that is likely to be observed, and limits statistical power (Wilson & Collins, 2019).

The second assumption is that parameters are stable over time (test–retest reliability). Importantly, parameter values that vary substantially over time within an individual limit the power to detect the effect of an intervention in a repeated-measures design (Pike et al., 2024). Many computational psychiatry applications, particularly in ‘experimental medicine’ (such as the designation of intermediate endpoints for clinical trials, mechanistic studies to investigate the changes to parameters following interventions, or studies to screen candidate interventions) thus require high test–retest reliability.

The third assumption is that parameters generalize across task contexts. This is a practical consideration that relates to a theoretical one: construct validity, or whether parameters index real constructs (and are not overfit, simply reflecting noise). This assumption is inherent in discourse surrounding the rationale for using computational models over model-agnostic measures: computational parameters are proposed to represent generative processes, and thus describe the way that cognition works, rather than being purely descriptive of behavioral outcomes (Adams, Huys, & Roiser, 2016; Haines et al., 2025). This is particularly salient when parameters are assigned the exact same name – for instance, learning rate – in different models. This name provides an explicit semantic link between parameters, and raises the question as to whether these parameters represent a latent generative cognitive process.

The fourth assumption is that parameters are related to symptoms (external, specifically clinical, validity). This is an important index of the success of computational psychiatry – which attempts to identify latent cognitive variables that may explain or predict the development of mental health symptoms. Some early computational psychiatry findings have failed to replicate, indicating that parameter-symptom relationships are not necessarily robust (Satti et al., 2024; Suddell et al., 2024; Vrizzi et al., 2025).

In this paper, we examine whether these four assumptions hold in a large online sample of participants performing commonly used computational psychiatry tasks. We focus on tasks that are thought to capture aspects of cognition relevant to mood and anxiety disorders – including reward and punishment learning (Pike & Robinson, 2022), risk and loss aversion (Charpentier, Aylward, Roiser, & Robinson, 2017; Giorgetta et al., 2012; Maner et al., 2007), reward bias (Pizzagalli et al., 2008), effort sensitivity (Bonnelle et al., 2015; Fleming, Robinson, & Roiser, 2023; Treadway et al., 2009; Valton et al., 2025), and exploration (Fan, Gershman, & Phelps, 2023). We fit behavior on each of these tasks with standard models reported in the computational cognitive modelling literature, and assess a) parameter recovery, b) test–retest reliability of parameters over two weeks, c) relationships between conceptually similar parameters across different tasks (generalizability); and d) relationships between parameters and mental health symptoms.

## Methods and materials

This study and its hypotheses were preregistered on the Open Science Framework, where the reader can also find open data and code (https://osf.io/6ug9m/). This OSF page links to two GitHub repositories, one which contains all the code to run these tasks within Gorilla, and one which contains all the models mentioned in this paper. Anonymized open data is provided within this OSF page, along with scripts for preprocessing, modelling, statistical analysis, and visualization.

### Participants

We recruited 548 participants through the Prolific platform (www.prolific.co; Palan & Schitter, 2018). Participants were a community-based sample aged between 18 and 80 years old, residing in the UK, who had indicated that they had never experienced mild cognitive impairment or dementia. Ethical approval was granted through the University College London Research Ethics Committee (ref. 6199/001). Participants were provided with an experiment briefing, consent forms, and contact information for the principal investigator, and were paid for participation at a rate of £7.50 per hour, plus bonus payments for task performance.

A subsample of participants who completed the initial study were re-contacted and asked to complete the battery of tasks and questionnaires again (for test–retest analyses). These participants were selected using a ‘custom allowlist’ on Prolific – into which we entered all participants whose data met basic quality criteria, i.e. who did not skip trials or respond with only a single response key.

## Procedure

Following consent, nine questionnaires and five tasks were administered through the online host platform Gorilla (www.gorilla.sc). Each experimental session lasted just over 1 hour (mean (*M*) = ~70 minutes) and ended with debriefing information. Bonus payments were distributed for the effort task by taking the average of the accumulated reward (*M* = £1.28), bonus payments for all other tasks were distributed with a mean of £0.80 and a standard deviation of £0.40 based on the total accumulated reward for each task.

### Materials

#### Questionnaires

Questionnaires measured various aspects of mental health relevant to depression, apathy and anhedonia, including the Spielberger State–Trait Anxiety Inventory (STAI-S and STAI-T; Spielberger et al., 1983), Temporal Experience of Pleasure Scale (TEPS; Gard, Gard, Kring, & John, 2006), Self-Rating Depression Scale (ZUNG; Zung, 1986), Apathy Evaluation Scale (AES; Marin et al., 2017) and Fatigue Severity Scale (FSS; Krupp, LaRocca, Muir-Nash, & Steinberg, 1989). In addition, we administered the Obsessive-Compulsive Inventory-Revised (OCI-R; Foa et al., 2002), which we predicted would minimally overlap with symptoms of depression and anxiety, as a positive control to establish discriminant validity. Other questionnaires collected were not analyzed here, and are listed in the Supplementary Materials.

#### Tasks

**Figure 1.**
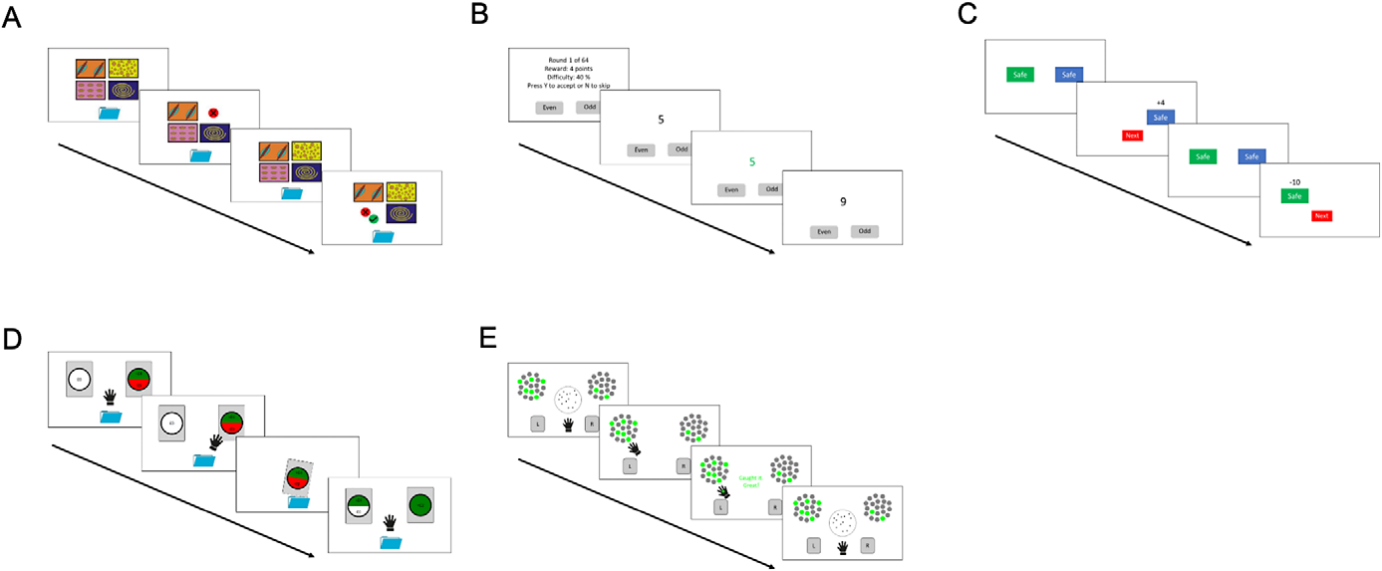
Diagrams of the five tasks participants completed. (a) Fluctuating bandit task. Multi-armed bandit tasks are classic reinforcement-learning paradigms, in which various stimuli are presented simultaneously and participants must choose between them. These stimuli are associated with reward and/or punishment, so each choice results in reinforcement, which participants can learn from. In this variant, the probabilities of reward and punishment fluctuate randomly over time, which requires learning to continue throughout the task. These tasks have been frequently used to examine whether there are systematic learning biases in depression and anxiety (Pike & Robinson, 2022). In our version of the task, participants completed 200 trials. On each trial, they were presented with four random ‘doors’ with an unknown probability of reward or punishment. The selected option produced an outcome of a loss (an image of a red cross), a gain (an image of a green tick), both, or nothing, before the next trial. The probability time series of rewards and punishments were orthogonal; thus both a gain and loss or neither could result from a given choice. The probability of receiving each reinforcement type for each option changed over the trials. Each box shows an example screen: screens one and three show the start of two different trials, where all four doors are presented. Screens two and four show two example outcomes – a loss (screen two) and a gain and loss together (screen 4). (b) Effort task. Previous research has investigated the willingness of individuals to exert physical effort, and related this individual difference to mental health constructs such as apathy and anhedonia (Bonnelle et al., 2015; Treadway et al., 2009; Valton et al., 2025). However, cognitive effort (Westbrook & Braver, 2015) may be more relevant in the domain of mental health. Alterations in the willingness to exert effort to obtain reward may represent impairments in reward valuation, effort valuation, or ability to balance benefits and costs. We used a task that manipulates cognitive effort without confounding it with task difficulty (Fleming, Robinson, & Roiser, 2023). At the start of each trial (screen 1), participants were told the level of effort and the reward associated with successful completion, and they could choose to ‘accept’ and perform the trial, or ‘reject’ the offer and move on to the next trial. If they ‘accepted’, they were presented with digits that they had to classify as even or odd within an individually calibrated time limit. Effort level was varied by manipulating the number of switches between even and odd in the sequence, and how often the same number was repeated. 20% difficulty involved trials with one or two switches, 40% three or four, 60% five or six, and 80% seven or eight. Reward values ranged from 1 to 4 points, which were paid as bonus payments on completion of the study. If more than two numbers within a sequence were incorrectly categorized, or participants took too long, no points were awarded. Numbers turned green when correct (screen 3), or red when incorrect, before the next number was presented. If the participant took too long, a message appeared to let them know and this number was skipped. Participants completed 12 practice trials, during which the time limit was individually calibrated, then 64 experimental trials. (c) Explore task. Within reinforcement learning, a fundamental question is how to balance the exploitation of known rewarding stimuli with exploration of new options which may provide (even better) rewards – the ‘explore/exploit’ trade-off (Addicott et al., 2017). The task we used was based on work using a multi-armed bandit paradigm to quantify different forms of uncertainty and examine how they influenced directed and random exploration (Gershman, 2018). Individual differences in these types of exploration may represent a promising transdiagnostic target (Lloyd et al., 2024): particularly given links between performance on this task and dopamine (Gershman & Tzovaras, 2018), a neurotransmitter implicated in depression, apathy, and anhedonia (Costello et al., 2023; Husain & Roiser, 2018). Additionally, differences in performance on this paradigm have been reported in anxiety disorders (Fan, Gershman, & Phelps, 2023). On each trial in our version of this task, participants were presented with two slot machines labeled risky or safe, which had unknown probabilities of reward or punishment. Participants had to learn over time, through feedback, which option was more likely to lead to a reward. The probabilities of reward changed over blocks of trials. ‘Safe’ options meant that the reward was fixed, and ‘risky’ options meant that the reward was taken from a probability distribution. The task had four conditions based on the type of options presented: safe-safe, safe-risky, risky-risky, and risky-safe, corresponding to left and right button options on the screen. This allowed us to observe how relative and total uncertainty affected the choice probability function (Gershman, 2018). This task involved 30 blocks of 10 trials (six blocks of safe-safe, eight blocks of each other condition). (d) Gamble task. Models of economic decision-making differentiate between risk aversion (a preference for certain over risky outcomes, even given the same expected value) and loss aversion (a tendency to weigh potential losses more than gains) (Kahneman & Tversky, 1979). These preferences may differ in anxiety disorders (Charpentier et al., 2017). In this task, participants chose between one safe (certain, shown on the left in this figure) and one risky (probabilistic, shown on the right) option on each trial. If a certain option was selected, participants received that amount deterministically. If participants chose the ‘risky’ option, they could receive either of the amounts shown, with a 50/50 probability. Our task involved 15 mixed trials (fixed amount versus binary gamble for loss or gain), 15 gain-only trials (fixed gain versus binary gamble for gain or £0), and 15 loss-only trials (fixed loss versus binary gamble for loss or £0). The value of the safe option (ranging from £1–3) was not always matched to the expected value of the gamble (ranging from £0–6). (e) Reward bias task. This task is a modified version of the probabilistic reward bias task, which has been used to investigate whether anhedonia is related to reward sensitivity (Pizzagalli et al., 2008). In this task, the original perceptual discrimination stimuli were replaced by random dot kinematograms (RDKs), to remove the potential confound of presenting emotional faces in the original task. On each trial, participants chose between two ‘slot machines’. Their choice was informed by the presentation of an RDK: the direction of motion of the majority of the dots indicated which of the two slot machines was ‘plugged in’. They responded using the L and R buttons. The slot machine on the side corresponding to the dot motion had a probabilistic chance of delivering a reward (green ball), with the probability shown visually by the ratio of green to grey balls. Choosing the other machine resulted in no reward. The reward probability of one machine was 30% (rich) whereas the other was 10% (lean). After receiving a reward (green ball) or no reward, the next trial began. The RDK could have five levels of coherence, which were alternating across trials, as manipulating difficulty allowed for better separation of reward bias from sensitivity. The learning component of the task was removed by explicitly informing participants which stimulus had higher reward probability, meaning that the bias was not learnt over time. This task included 100 trials, 40 of which had the potential to result in a reward. Figure 1. long description.

Participants completed five cognitive tasks (see Figure 1).

### Analysis

#### Computational modeling

We fit separate families of five computational models to each of the five tasks (code is available on the OSF, as noted above; and equations are available in the Supplementary Materials). Models were constructed using a hierarchical Bayesian approach – with group- and individual-level priors. Estimation was performed using individual-Markov Chain Monte Carlo (MCMC) sampling in Stan (implemented using cmdstanr, v0.6.0.9000 locally, or v0.8.1 on a high-performance compute cluster). Each model was fit using four chains, each with 2000 samples (the first 1000 of which were discarded as burn-in samples). After model-fitting, we assessed sampling performance using the Gelman-Rubin statistic (Vehtari et al., 2021), where values above 1.01 are considered to represent poor convergence. Additionally, we examined trace plots of the log posterior to check whether the chains were well-mixed.

The winning model was then identified using the integrated Bayesian Information Criterion (Huys et al., 2011), which incorporates a penalty for complexity.

*Assumption 1: Parameter recovery.* To examine parameter recovery, we used the parameters estimated from the best-fitting computational model of each task (‘known parameters’) and then ran the model forward to generate ‘synthetic data’. This synthetic data was then re-fit to the best-fitting computational model, producing ‘recovered parameters’, which were correlated with the known parameter values. A high correlation indicates that the model shows good recovery of parameter values – i.e. that parameter values are likely to be estimated consistently.

*Assumption 2: Parameter stability over time.* To examine the test–retest reliability of parameters, we calculated both ‘absolute agreement’ and ‘consistency’ intraclass correlation coefficients (ICC(A,1) and ICC(C,1), respectively). We used (and report) two different approaches to fitting models over two sessions: 1. Using two separate priors and fitting models separately to each session, 2. Explicitly estimating a correlation matrix within the model (Haines et al., 2025).

*Assumption 3: Parameter generalizability across task contexts.* We analyzed relationships between parameters that, at least in principle, measure the same cognitive construct across different tasks in several ways. First, we examined relationships between pre-registered groups of parameters – learning rates and inverse temperatures/sensitivity parameters – using Pearson’s correlations. Then, we estimated an ‘upper bound’ for these relationships: we took the strongest relationship between two parameters in different tasks, and fitted these two tasks within one model, explicitly estimating the correlation matrix between the parameters of interest in the two tasks. We then performed an exploratory correlation analysis, and related all task parameters to all other task parameters. We also ran an exploratory factor analysis to identify whether there were any natural groupings of parameters, and validated this structure using a confirmatory factor analysis (using the ‘lavaan’ package in R) on the test–retest data.

*Assumption 4: Symptom-parameter associations.* To reduce the dimensionality of our mental health questionnaire data we performed an item-level exploratory factor analysis, using a well-established approach in the field (Gillan et al., 2016; Wise, Robinson, & Gillan, 2023).

We then took a similar approach to prior work (Moutoussis, Hopkins, & Dolan, 2018) to test this assumption: we initially analyzed pre-registered relationships, which included latent mental health variables derived using the EFA above, and constructs reflected in single questionnaires. For significant relationships, we estimated an ‘upper bound’ on these by estimating these relationships within the model (Moutoussis, Hopkins, & Dolan, 2018), mirroring our approach to analysis for test–retest reliability and context-generalization. We then performed an exploratory analysis, estimating all relationships between all parameters and mental health latent variables and questionnaire sum scores.

## Results

### Participants

We included 548 participants in the main analyses, of whom 326 were female, 214 were male, and 8 preferred not to say. See Table 1 for more demographic details and a summary of symptom questionnaire scores. Due to a technical error, 47 participants received no rewards on the reward bias task and therefore their data were excluded. One participant’s data were removed from the bandit task as they only responded using one key. The rest of these participants’ data (the remaining tasks) was included.

**Table 1.** Demographic variables with mean and standard deviation for both the main sample (*n* = 548) and the retest sample (*n* = 115) Table 1. long description.

Variable	Main sample (*n* = 548)		Retest sample (*n* = 115)	
	Mean	SD	Mean	SD
Age	36.5	13.3	38.3	14.1
Prolific score	99.3	1.39	99.5	1.2
FSS score	35.4	12.6	36.2	11.8
AES score	35.6	9.0	35.7	8.6
Zung score	44.2	10.1	42.2	11.2
STAI-S score	44.1	11.2	39.6	12.8
STAI-T score	46.5	12.9	46.3	13.8
OCI-R score	18.0	12.0	18.1	12.3
TEPS score	48.6	10.9	46.8	11.0

*Note:* FSS, Fatigue Severity Scale; AES, Apathy Evaluation Scale; Zung Score, Zung Self-Rating Depression scale; STAI, Spielberger State–Trait Anxiety Inventory, either version T (Trait) or S (State); OCI-R, Obsessive-Compulsive Inventory – Revised; TEPS, Temporal Experience of Pleasure Scale.

One-hundred and fifteen participants completed the same tasks after 2 weeks, of whom 63 were female and 51 were male.

### Assumption 1: Parameters are recovered reliably

**Figure 2.**
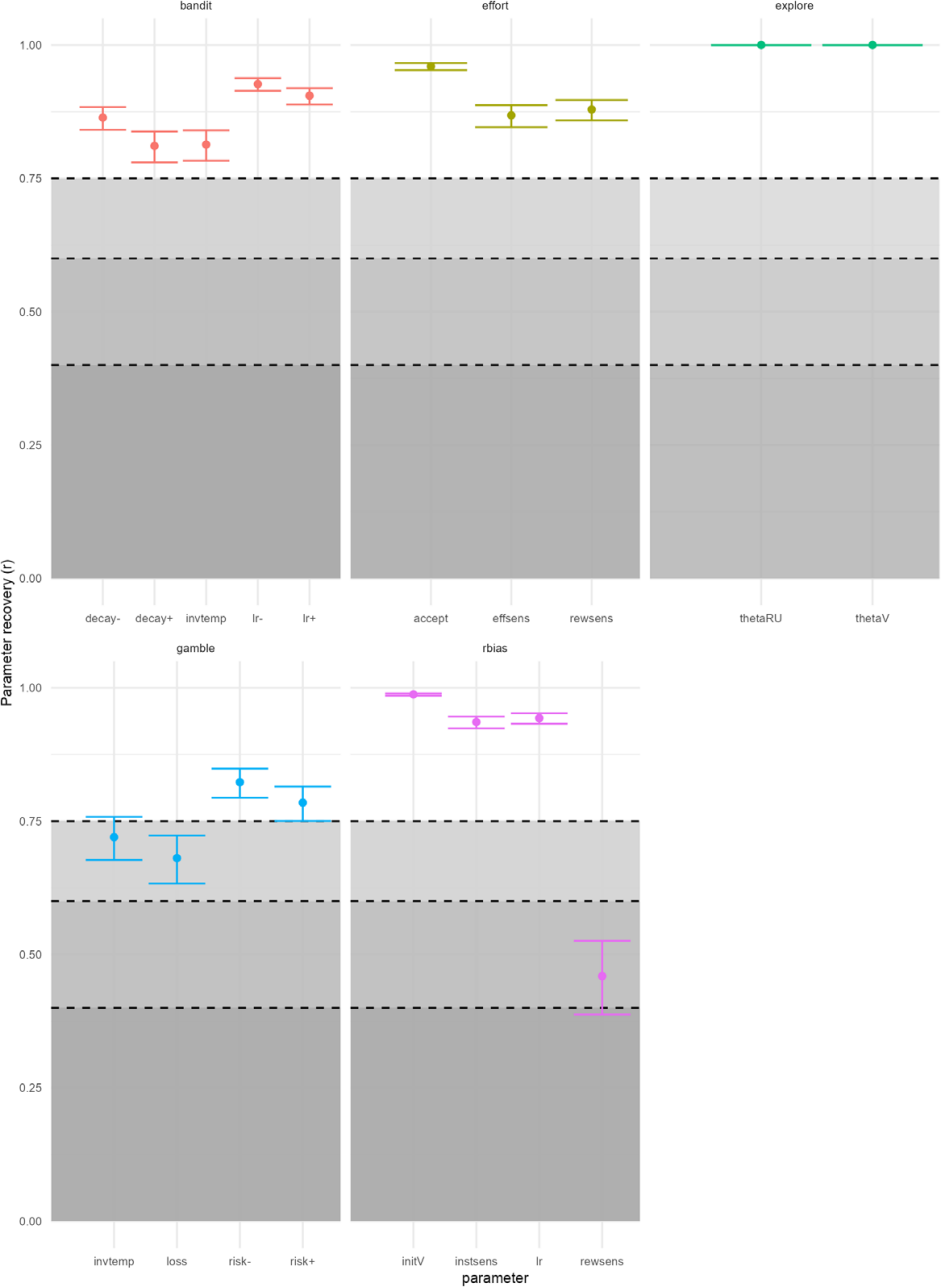
Recovery of task parameters. Each point is the point estimate of a Pearson’s correlation between originally estimated parameters and parameters recovered after synthetic data was generated using these fitted parameter values. Error bars represent the 95% confidence interval on the correlation estimate. All correlations were statistically significant. Note that the explore task model does not require fitting; hence, there is an exact analytic solution and the recovery is perfect (correlation of *r* = 1.0). Shaded areas between dashed lines indicate correlations suggestive of poor recovery (<0.4), fair recovery (0.4–0.6), good recovery (0.6–0.75) and excellent recovery (0.75–1.0). Parameter names are abbreviated: ‘lr’ is learning rate (‘lr+’ is a reward learning rate, and ‘lr−’ is punishment learning rate); ‘decay+’ is decay of positive values, and ‘decay−’ is decay of negative values; ‘sens’ is sensitivity (‘rewsens’ is reward sensitivity, ‘effsens’ is effort sensitivity, and ‘instsens’ is instruction sensitivity); ‘invtemp’ refers to inverse temperature; ‘acc’ is acceptance bias in the effort task; ‘loss’ is loss aversion, ‘risk+’ is risk aversion on gain trials, and ‘risk−’ is risk aversion on loss trials of the gamble task; ‘thetaV’ is value difference, and ‘thetaRU’ is relative uncertainty in the explore task; finally, ‘initV’ represents ‘initial value’ in the reward bias task. Figure 2. long description.

For all of our best-fitting models (see Supplementary Material for a summary of the model comparison), parameters were generally recovered with good or higher reliability (Cicchetti, 1994). The exception to this was reward sensitivity in the reward bias model, which was recovered with approximately *r* = 0.459 (fair reliability). Several other parameters fell in the ‘good’ recoverability bracket (the lambda parameter in the gamble task, tau in the gamble task) but all others showed excellent recovery (Figure 2).

### Assumption 2: Parameters are generally stable over time

**Figure 3.**
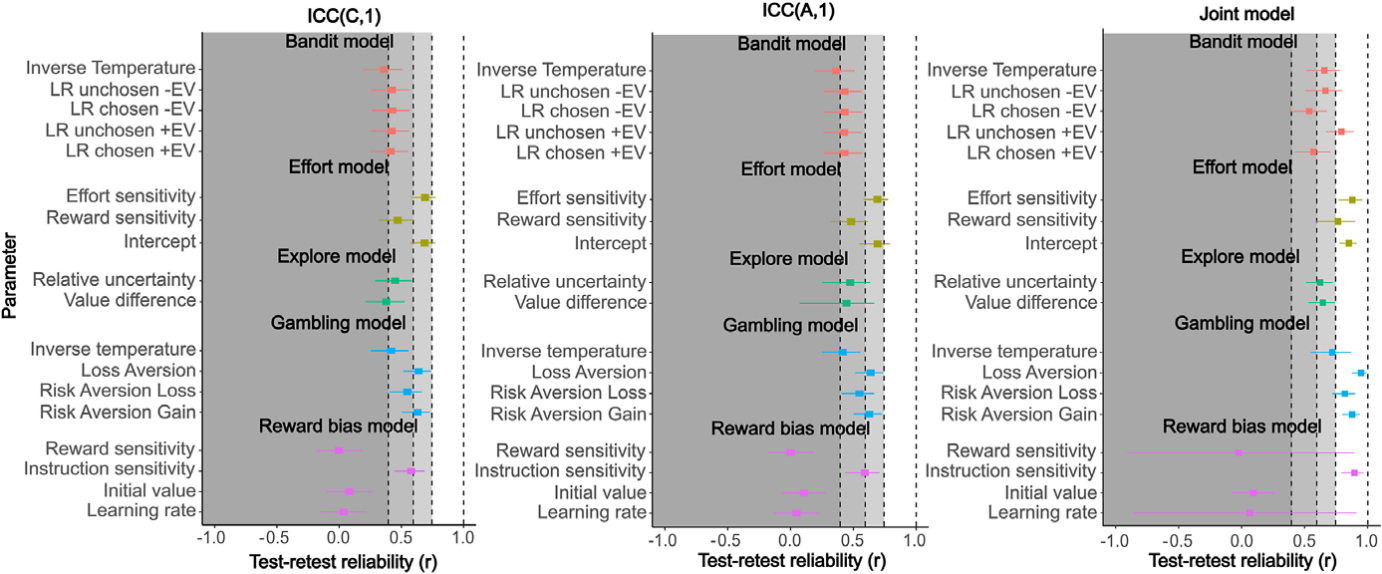
Test–retest reliability. Each plot shows the mean and 95% confidence interval for the test–retest reliability of each parameter from the best-fitting model. ICC(C,1) refers to consistency intraclass correlation coefficient, ICC(A,1) to absolute ICC, and ‘joint model’ refers to the correlation coefficient estimated by embedding the correlation matrix within the model. Shaded areas between dashed lines indicate correlations suggestive of poor reliability (<0.4), fair reliability (0.4–0.6), good reliability (0.6–0.75) and excellent reliability (0.75–1.0). Figure 3. long description.

The majority of model parameters showed fair or better test–retest reliability when reliability was calculated using point estimates of parameter values. In Figure 3 (see also Supplementary Table 2), we display ICC(C,1) (left panel), ICC(A,1) (middle panel), and correlations estimated within models fit to both session 1 and session 2 jointly (right panel). ICC(C,1) reflects consistency (i.e. reliable rank order), whereas ICC(A,1) reflects absolute agreement (i.e. reliable numeric values, including mean). The joint model correlations increase the estimates of test–retest reliability, such that the majority of parameters show ‘excellent’ reliability (from a mean of 0.426 to a mean of 0.635). The exception is the reward bias task, where three out of four parameters were estimated to have test–retest reliability of zero, regardless of the method used.

### Assumption 3: Conceptually related parameters do not generalize robustly across task contexts

#### Pre-registered analyses

**Figure 4.**
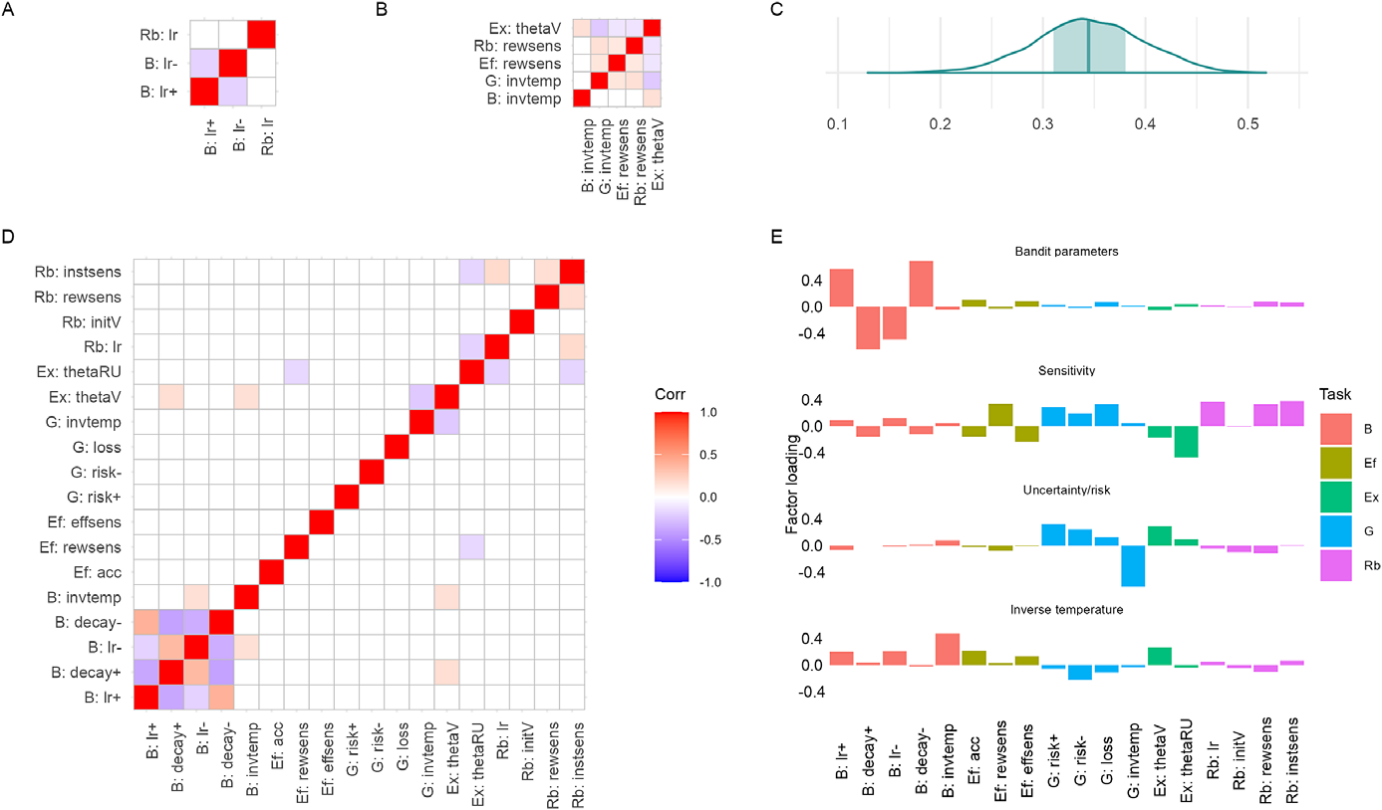
Relationships between parameters. (a) The correlation matrix of the relationship between parameters that were anticipated to reflect a ‘learning rate’ construct. (b) The correlation matrix of the relationship between parameters that were anticipated to reflect an ‘inverse temperature’ construct. Colors represent strength of correlation. Relationships that did not reach statistical significance are shown in white. (c) We estimated an ‘upper bound’ for the parameter-parameter relationship by embedding a correlation matrix within the model. The figure shows a central (quantile-based) posterior interval estimate from the MCMC draws, showing the 95% mass interval shaded, with the median as a solid line in the centre. (d) Exploratory analyses of all possible parameter-parameter relationships (Bonferroni-corrected for multiple comparisons, only significant correlations shown). Note that the significant relationships did not change when correcting for age and gender, except that there was a relationship between the reward bias reward sensitivity parameter and the gamble inverse temperature parameter. (e) The result of an exploratory factor analysis of the parameters across all tasks: a four-factor structure was found, with some ‘parameter constructs’ represented as factors. However, this structure had less evidence than a factor structure in which parameters were grouped by task, in a confirmatory factor analysis on task data from time 2. Tasks are described using a letter or pair of letters: ‘B’ for bandit, ‘Ef’ for effort, ‘Ex’ for explore, ‘G’ for gamble, and ‘Rb’ for reward bias. Parameters are also abbreviated as above: ‘lr’ is learning rate (‘lr+’ is a reward learning rate, and ‘lr−’ is punishment learning rate); ‘decay+’ is decay of positive values, and ‘decay−’ is decay of negative values; ‘sens’ is sensitivity (‘rewsens’ is reward sensitivity, ‘effsens’ is effort sensitivity, and ‘instsens’ is instruction sensitivity); ‘invtemp’ refers to inverse temperature; ‘acc’ is acceptance bias in the effort task; ‘loss’ is loss aversion, ‘risk+’ is risk aversion on gain trials, and ‘risk−’ is risk aversion on loss trials of the gamble task; ‘thetaV’ is value difference, and ‘thetaRU’ is relative uncertainty in the explore task; finally, ‘initV’ represents ‘initial value’ in the reward bias task. Figure 4. long description.

To investigate whether conceptually related parameters generalize across task contexts, we preregistered that we would compare learning rates and inverse temperatures across tasks. Whilst only two of the tasks included learning rate parameters (bandit and reward bias tasks), there were several models that contained a parameter that plays a similar role to ‘inverse temperature’ or ‘reward sensitivity’, in scaling either reward values or the difference between them. The results of these analyses are shown in Figure 4a,b, and in Supplementary Table 3 – which also shows that these relationships do not change when controlling for age and gender. As when testing the test–retest reliability, we also estimated the ‘upper bound’ of this relationship by estimating this relationship within the model, and explicitly embedding a covariance matrix – accounting for sample-by-sample noise and using the variance information of the full posterior distribution, rather than just a single midpoint estimate. When testing this with the two most correlated inverse temperatures (from the bandit and explore tasks: *r*_546_ = −0.227, *p* < 0.001), the strength of the relationship increased (*mean r* = 0.345, *SD* = 0.0529 [95%CI: 0.258, 0.432]; note that the parameter in the explore task was inverted for this analysis so the relationship is in the positive direction; plot of central posterior interval estimate from posterior draws shown in Figure 4c). This improvement still indicates only a moderate relationship between these parameters, and implies that measurement noise is limiting our ability to detect these relationships.

#### Exploratory analyses

We also correlated all parameters with each other (Figure 4d, Bonferroni-adjusted p-value of 0.05/153). There were some significant relationships between parameters from different tasks that survived Bonferroni correction for multiple comparisons. These included several of the relationships listed above between ‘inverse temperature’-like parameters (explore value difference and bandit inverse temperature, gamble inverse temperature and explore value difference), bandit decay positive and explore value difference (*r*_545_ = 0.156, *p* < 0.001), effort reward sensitivity and explore relative uncertainty (*r*_546_ = −0.176, *p* < 0.001), reward bias learning rate and explore relative uncertainty (*r*_499_ = −0.190, *p* < 0.001), reward bias instruction sensitivity and explore relative uncertainty (*r*_499_ = −0.184, *p* < 0.001), as well as relationships between several parameters in the reward bias task.

We subsequently performed an exploratory factor analysis on *z*-scored parameters to understand whether there was a latent structure underpinning parameters found in different tasks. Parallel analysis and a scree plot suggested four factors, which are shown in Figure 4e. The factors that emerge seem to reflect a separate factor for inverse temperature, and perhaps one for other types of sensitivity (to reward, effort, and uncertainty). There does not appear to be a factor specific to learning rate. When the factor structure obtained by this EFA was tested using confirmatory factor analysis on parameters obtained from the test–retest data, this factor structure did not fit the data better than a model in which parameters from each task loaded onto their own separate factor (BIC = 5192.3 versus 5920.0, AIC = 5786.0 versus 5804.7, CFI = 0.702 versus 0.583, RMSEA = 0.063 vs 0.073, SRMR = 0.083 versus 0.089, χ^2^ = 181.929 versus 208.68). It is worth noting that neither of these models meets conventional standards for a good fit.

### Assumption 4: Parameters relate weakly to symptoms

We performed dimensionality reduction on the seven main questionnaires of interest, using EFA. Visual inspection of scree plots resulted in a four-factor solution (Supplementary Figure 4), which we named according to the items that loaded most strongly onto those factors: ‘anxiety/low mood’, ‘motivation/interest’, ‘fatigue’ and ‘compulsive/intrusive thought’.

#### Pre-registered analyses

We pre-registered several hypothesized relationships between parameters and EFA-derived mental health constructs – though these were limited to the level of constructs as we did not know which models would best fit the data, nor what specific factor structure would emerge from our EFA, (*Note* that here we use the ‘anxiety/low mood’ factor and the Zung Self-Report Depression Scale to test hypotheses using the ‘depression’ construct, the ‘motivation/interest’ factor and Temporal Experience of Pleasure scale to represent anhedonia, and the ‘motivation/interest’ factor and Apathy Evaluation Scale to represent apathy. This is based on an interpretation of the ‘motivation/interest’ factor as representing both apathy and anhedonia together, given that this is largely composed of items from both the AES and the TEPS, though one could argue that the ‘fatigue’ factor may also be related to apathy. See the Supplementary Materials for more details on the EFA.) These comparisons are not Bonferroni-corrected. Supplementary Table 4 shows these results, and that the inference does not change when controlling for sex and gender.

**Figure 5.**
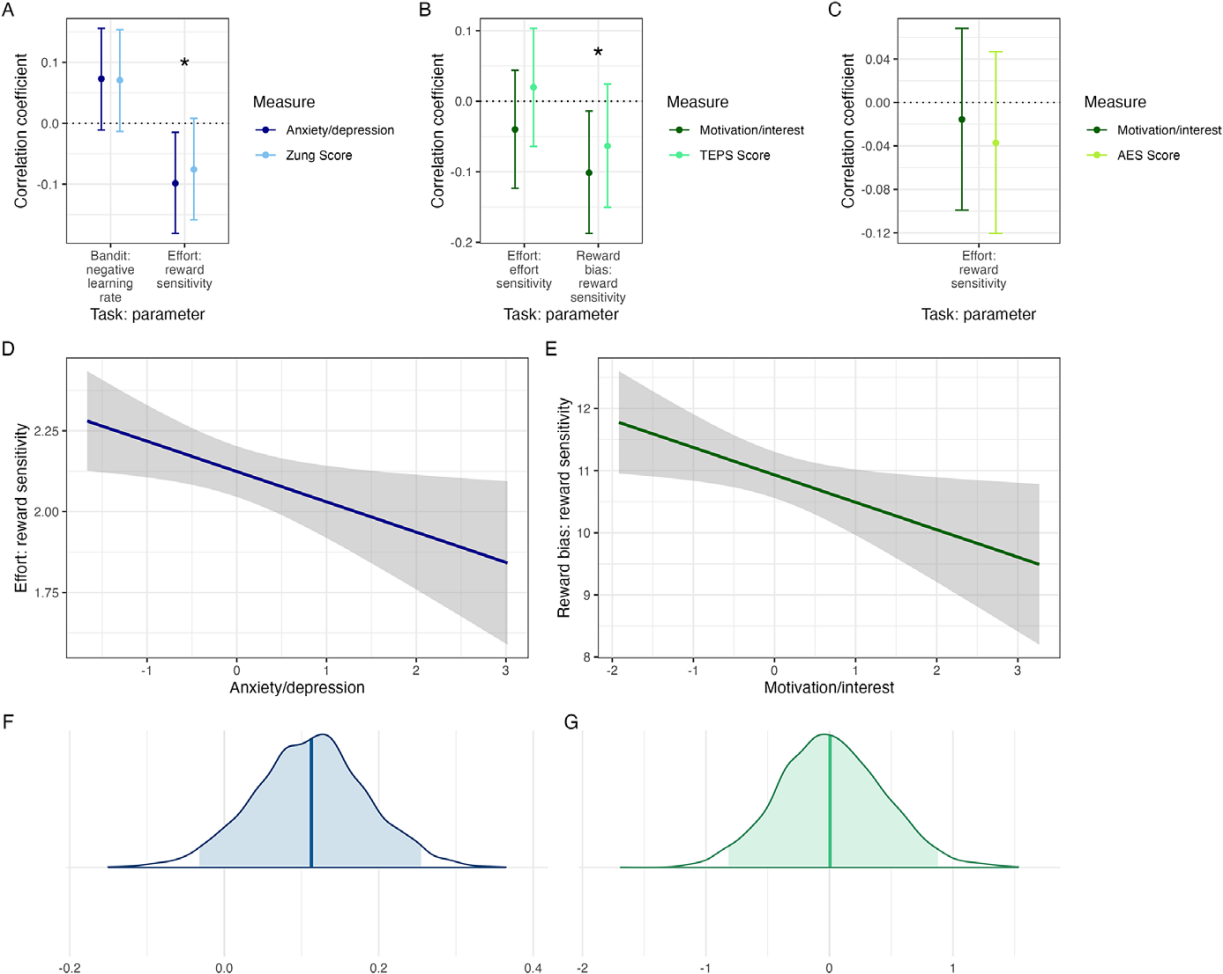
*A priori* predicted relationships between parameters and clinical measures. (a–c) Correlation coefficients and 95% confidence intervals of relationships between parameters and either EFA latent variables, or questionnaire totals. (d–e) Relationships and confidence intervals for significant relationships from a–c. (f–g) Relationships when these were estimated by embedding the effect within the generative model – this is a central (quantile-based) posterior interval estimate from the MCMC draws, showing the 95% mass interval shaded, and the median as a solid line in the centre. Figure 5. long description.

*Depression.* We hypothesized that higher levels of depressive symptoms would correlate with greater punishment learning rate in the 4-armed bandit task. This hypothesis was not supported using the first factor from our EFA (*r*_545_ = 0.073, *p* = 0.088, Figure 5a), or using the Zung Self-Rated Depression Scale (*r*_545_ = 0.071, *p* = 0.099, Figure 5a) – although we note that both these correlations only narrowly missed statistical significance.

We also hypothesized that depressive symptoms would correlate with lower reward sensitivity in the effort-based decision-making task: this was supported using the anxiety-depression factor (*r*_546_ = −0.0984, *p* = 0.02115, Figure 5a,d) but not the Zung Scale (*r*_546_ = −0.0756, *p* = 0.0769, Figure 5a), which narrowly missed statistical significance. This relationship was slightly strengthened when estimated within the generative model, *𝛽* = 0.111, *SD* = 0.0735 (Figure 5f).

*Anhedonia.* We predicted that a higher anhedonia score would correlate with greater effort sensitivity in the effort-based decision-making task, which was not supported using either the motivation factor (*r*_546_ = −0.0399, *p* = 0.3507) or the TEPS scale (*r*_546_ = 0.0197, *p* = 0.6488; Figure 5b).

We predicted that anhedonia would be related to lower reward sensitivity in the reward bias task, which was supported using the motivation/interest factor (*r*_499_ = −0.101, *p* = 0.0233, Figure 5b,e) but not using the TEPS questionnaire (*r*_499_ = −0.063, *p* = 0.1565, Figure 5b). This relationship was weaker and non-significant when estimated within a generative model: *β* = 0.016, *SD* = 0.427 (Figure 5g), consistent with the poor recoverability of this parameter.

*Apathy.* Finally, we predicted that apathy would correlate with lower reward sensitivity in the effort-based decision-making task, which was also not supported using either the motivation/interest factor (*r*_546_ = −0.0156, *p* = 0.7155), or the AES (*r*_546_ = −0.03721341, *p* = 0.3846), see Figure 5c.

#### Exploratory analyses

**Figure 6.**
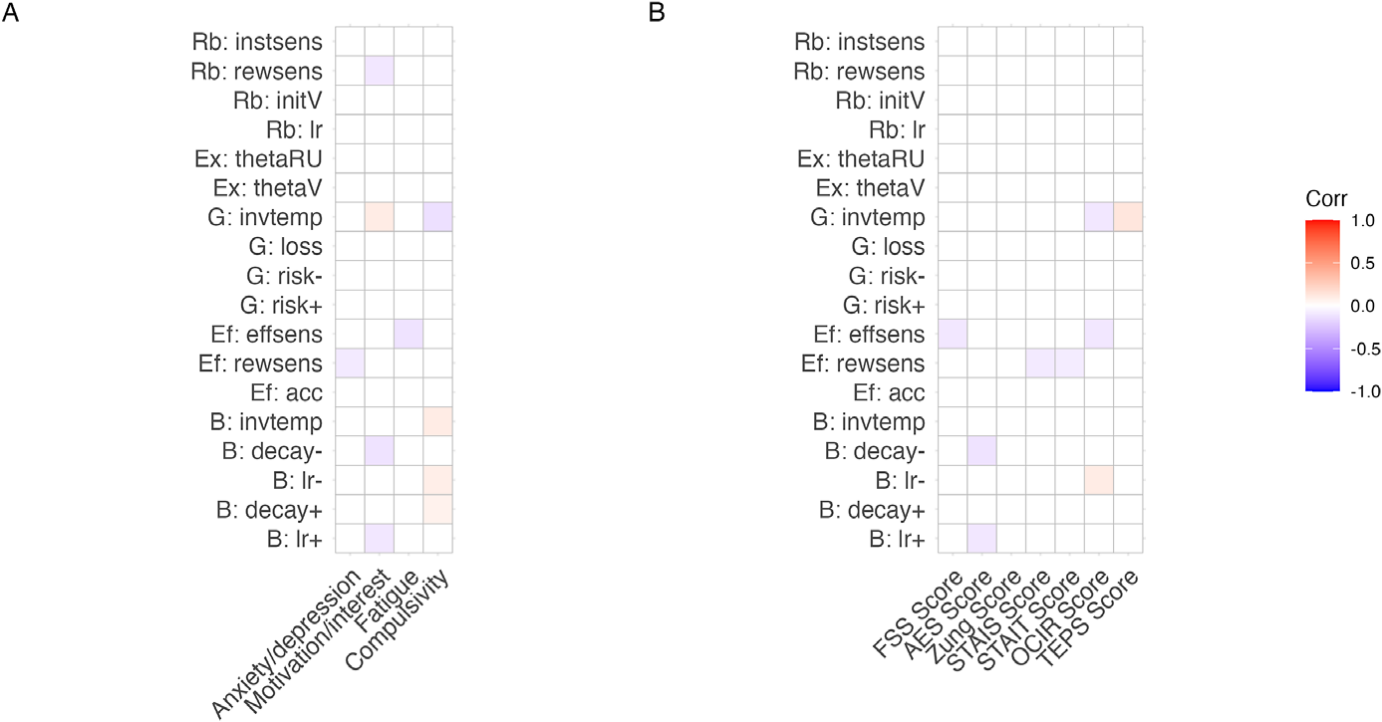
Correlations between clinical measures and parameters. (a) Correlations between all model parameters and all four factors from the exploratory factor analysis. (b) Correlations between the seven questionnaires completed and all model parameters. Color indicates the strength of the correlation. Cells are shown in white if they were not significant at the uncorrected *p* < 0.05 level. Note that adjusting for age and gender did not alter these results. As above, tasks are described using a letter: ‘B’ for bandit, ‘Ef’ for effort, ‘Ex’ for explore, ‘G’ for gamble, and ‘Rb’ for reward bias. Parameters are also abbreviated as above: ‘lr’ is learning rate (‘lr+’ is a reward learning rate, and ‘lr−’ is punishment learning rate); ‘decay+’ is decay of positive values, and ‘decay−’ is decay of negative values; ‘sens’ is sensitivity (‘rewsens’ is reward sensitivity, ‘effsens’ is effort sensitivity, and ‘instsens’ is instruction sensitivity); ‘invtemp’ refers to inverse temperature; ‘acc’ is acceptance bias in the effort task; ‘loss’ is loss aversion, ‘risk+’ is risk aversion on gain trials, and ‘risk−’ is risk aversion on loss trials of the gamble task; ‘thetaV’ is value difference, and ‘thetaRU’ is relative uncertainty in the explore task; finally, ‘initV’ represents ‘initial value’ in the reward bias task. Figure 6. long description.

Correlation analyses between all four factors and all parameters identified some weak relationships, though none survived Bonferroni correction. These are shown in Figure 6a. Similarly, correlation analyses between all questionnaire scores and all parameters identified weak relationships that did not survive Bonferroni correction (Figure 6b).

## Discussion

In this study, we tested several assumptions that are fundamental to, but often go unexamined in, computational psychiatry research. Specifically, we examined parameter recovery, test–retest reliability, parameter generalizability across task contexts, and the relationships between computational parameters and mental health constructs. While we found generally excellent parameter recovery and good test–retest reliability, there was limited evidence that there were any relationships between computational parameters from different reward processing tasks ostensibly measuring similar constructs, and limited evidence for relationships between these parameters and measures of anxiety or depression.

We found high parameter recovery for the majority of task models. This is reassuring, as recovery is likely to provide an upper limit on other related quantities such as test–retest reliability, but is often not reported despite its recognized importance for clinical applications (Browning et al., 2020). We note that ‘reward sensitivity’ in the reward bias task had poor recovery, suggesting further optimization to this task or model is required, in a similar vein to work improving test-retest reliability (Brown et al., 2020).

Generally fair test–retest reliability for model parameters, which was similar to test–retest reliability of model-agnostic measures estimated using logistic regression (Supplementary Table 1), could be improved by estimating reliability within the model-fitting process. However, test–retest reliability did not improve for all model parameters (particularly for the reward bias model), which suggests that estimating the reliability within the model does not *necessarily* improve reliability, an important rebuttal to critics of this method.

Our finding of good-to-excellent reliability contrasts with other notable recent findings which did not use a hierarchical Bayesian approach (Vrizzi et al., 2025), but there is clearly room for improvement: indeed, there are many ongoing efforts to improve the reliability of computational parameters (Brown et al., 2020; Haines et al., 2025; Mkrtchian, Valton, & Roiser, 2023; Zorowitz et al., 2023; Zorowitz & Niv, 2022). Importantly, if parameters are being used to index a construct that is expected to fluctuate over a short timescale (e.g. daily mood), then high test–retest reliability is undesirable. However, fluctuations in this case should not be random but tightly correlated with the measure of mood (Schaaf et al., 2024) – the average within-person correlation between their mood and the parameter may therefore be more relevant, but estimating this would require a large amount of data per participant. More intensive longitudinal approaches have begun to characterize the dynamic processes that affect computational parameters – including mood/affect, practice, and noise – which may allow us to estimate test–retest reliability more precisely (Schurr et al., 2024).

The other two assumptions we tested (generalizability of conceptually related parameters, and relationships between parameters and symptoms) were less well supported in this dataset. We observed at best moderate relationships between conceptually similar parameters across different task contexts, and these were generally limited to inverse temperature-like parameters. Additionally, we found weak relationships between parameters and mental health constructs, but only in some tasks, with effect sizes on the order of *r* = 0.1.

There are several possible reasons for these assumptions not being fully met. First, these cognitive task models may measure meaningful constructs but imprecisely, meaning that we see small and inconsistent relationships both between different parameters and between parameters and mental health measures. Even when estimating relationships within the models themselves, thus allowing for partial pooling of estimates and accounting for sample-by-sample uncertainty (Brown et al., 2020), these relationships remained weak.

Second, the relationships between parameters and symptoms may be nonlinear. In this paper, we assumed a linear relationship between mental health symptoms and parameters. This reflects a growing trend in the field – of characterizing mental health as a continuum, with symptoms increasing linearly along with impairment. There is emerging evidence in support of this characterization (Conway & Krueger, 2021; Haslam, McGrath, Viechtbauer, & Kuppens, 2020), but some argue instead that there is some nonlinearity, or threshold, that separates ‘illness’ (or treatment seeking) from ‘wellness’ in mental health (Curtis & Derks, 2018). This might explain the difficulty in finding associations in the general population between continuously measured symptoms and cognition, compared to finding group differences between patients and controls. In such studies conducted in unselected samples, the majority of the variation in symptoms falls in the non-clinical range, which may inherently limit the magnitude of associations that can be expected.

Third, it is not only in the computational psychiatry domain that issues have emerged in relating self-report measures to behavior. Importantly, symptoms are measured on a different timescale to computational parameters. Symptoms are putatively measured over a period of weeks (or, in trait measures such as the STAI-T, over a lifetime), whereas parameters are expressions of choices made over a brief period (maximum half an hour). Self-report measures can also be biased – by the self-construct of the individual reporting, by demand characteristics of the study, or by inattention if scores on the instrument are skewed, owing to a preponderance of positively scored items (as is the case for many mental health questionnaires, Zorowitz, Solis, Niv, & Bennett, 2023). Symptoms are also heterogenous, so symptom scales may appear highly stable within-individuals, but differ in their meaning between individuals (e.g., anxiety is high in most mental health disorders, but the same symptom profile may not have the same underlying cause in patients with psychosis, depression, or autism). More broadly, the replicability of many results across psychology and neuroscience has been called into question – computational psychiatry can and should learn from the best practices and cautionary tales in mental health research in domains such as neuroimaging (Blair, Mathur, Haines, & Bajaj, 2022; DeYoung et al., 2025; Elliott et al., 2020; Gell et al., 2023; Hajcak, Meyer, & Kotov, 2017), animal neuroscience (Rosso et al., 2022; Völter, Tinklenberg, Call, & Seed, 2018), and artificial intelligence/machine learning (Adler et al., 2024; Gonzalez, Georgeson, & Pelham, 2024).

Several limitations of this study merit comment. We focus on one specific area of computational psychiatry research: depression and anxiety. The questionnaires were selected to index constructs relevant to depression: apathy, anhedonia, mood and anxiety, with one questionnaire selected to measure a separable construct (compulsivity) for discriminant validity. Within the field, some diagnoses and clinical phenomena may be more robustly associated with computational parameters than others (Wise, Robinson, & Gillan, 2023). Additionally, we focus on one type of model: algorithms that explain learning and decision-making. The majority of models included in this paper are reinforcement learning models, but many other (sometimes overlapping) frameworks exist (neural network models, biophysical models, predictive coding models, and Bayesian belief-updating models). The conclusions of this paper cannot be assumed to generalize to all of these approaches, but provide an example evaluation of these assumptions with respect to one highly studied area. Relatedly, only two of our tasks had a best-fitting model containing any learning rate parameters, limiting our ability to infer whether learning rates generalize. One of these tasks, the reward bias task, had poor parameter recovery and reliability – with recent evidence showing that other approaches to modelling this task, such as drift diffusion modelling, may produce superior psychometric performance (Dillon et al., 2024). Further, there are other assumptions made within computational psychiatry that we have not tested: including that tasks are sensitive and valid (Wilson & Collins, 2019), and that parameters are modifiable – some cognitive features of mental ill-health, such as many ‘cold cognition’ measures, seem to be so stable over time that they are not altered following remission of symptoms (Rock, Roiser, Riedel, & Blackwell, 2014), and may even pre-date the onset of symptoms (Halahakoon, Lewis, & Roiser, 2019). It is also important to highlight that this study was conducted online in an unselected sample, and our findings may not hold in clinical samples.

In this paper, we outlined four assumptions commonly made within computational psychiatry, and tested them in a specific set of tasks used frequently in the anxiety/depression literature. While we should take care to avoid overgeneralizing from these findings, there are a number of resulting recommendations for researchers in the field of computational psychiatry. First, consider including parameter recovery and test–retest reliability as criteria within model comparison. This may make results in this field more meaningful and reproducible. Notably, the reward bias model that won our model comparison had parameters that showed slightly worse test–retest reliability than another (model 5, see Supplementary Figure 3). If one was interested in a parameter that showed better test–retest reliability using model 5, that would be a sensible reason to use that as the chosen model. Second, optimize tasks, models, and fitting procedures for reliability (Brown et al., 2020; Zorowitz et al., 2023; Zorowitz & Niv, 2022). Third, where possible, embed inference within generative models. We employed this approach to estimate test–retest reliability, to estimate relationships between task parameters, and to estimate relationships between parameters and symptoms. In the majority of cases (but not universally), this increased the estimate of the relationship – likely because this approach incorporates and accounts for the variance of posterior samples, not just the mean.

In conclusion, we have presented a framework for testing the assumptions made, often implicitly, in computational psychiatry research, and examined them within the context of depression and reinforcement-based learning and decision making. We conclude that while parameters may show good recovery and test–retest reliability, they are not necessarily representing consistent latent variables such as ‘learning rate’ across task contexts, and nor are they strongly related to symptoms.

## Supporting information

10.1017/S0033291726105340.sm001 Pike et al. supplementary material 1 Pike et al. supplementary material

10.1017/S0033291726105340.sm002 Pike et al. supplementary material 2 Pike et al. supplementary material

## Supplementary material

The supplementary material for this article can be found at http://doi.org/10.1017/S0033291726105340.

## Long descriptions

### Figure 1. Long description

Panel A, Fluctuating bandit task. Four colored doors are shown in a grid. A sequence of four screens shows a trial start, a loss outcome indicated by a red cross, another trial start, and a combined gain and loss outcome indicated by a green tick and red cross.

Panel B, Effort task. Four screens show the trial sequence. Screen 1 displays text for Round 1 of 64, Reward 4 points, Difficulty 40 percent, and instructions to press Y or N. Screen 2 shows a digit 5 with Even and Odd buttons. Screen 3 shows the digit 5 in green indicating a correct response. Screen 4 shows a new digit 9.

Panel C, Explore task. Four screens show slot machines labeled Safe or Risky. The sequence shows a choice between two Safe machines, a reward outcome of plus 4 above a blue Safe machine with a red Next button, another choice between two Safe machines, and a loss outcome of minus 10 above a green Safe machine.

Panel D, Gamble task. Four screens show a choice between a certain outcome on the left and a 50/50 gamble on the right. The gamble is represented by a circle split into two colors. A hand icon indicates the selection process, leading to a final screen showing the outcome of the chosen option.

Panel E, Reward bias task. Four screens show a central R D K circle with moving dots between two slot machines represented by clusters of grey and green balls. Buttons labeled L and R are positioned below the machines. A hand icon selects a machine based on dot motion, followed by a feedback screen showing a green ball and the text Caught it Great.

Navigate back to Figure 1..

### Table 1. Long description

The table consists of five columns: Variable, Main sample (n = 548) Mean and S D, and Retest sample (n = 115) Mean and S D.

* Age: Main sample Mean 36.5, S D 13.3; Retest sample Mean 38.3, S D 14.1.

* Prolific score: Main sample Mean 99.3, S D 1.39; Retest sample Mean 99.5, S D 1.2.

* F S S score: Main sample Mean 35.4, S D 12.6; Retest sample Mean 36.2, S D 11.8.

* A E S score: Main sample Mean 35.6, S D 9.0; Retest sample Mean 35.7, S D 8.6.

* Zung score: Main sample Mean 44.2, S D 10.1; Retest sample Mean 42.2, S D 11.2.

* S T A I-S score: Main sample Mean 44.1, S D 11.2; Retest sample Mean 39.6, S D 12.8.

* S T A I-T score: Main sample Mean 46.5, S D 12.9; Retest sample Mean 46.3, S D 13.8.

* O C I-R score: Main sample Mean 18.0, S D 12.0; Retest sample Mean 18.1, S D 12.3.

* T E P S score: Main sample Mean 48.6, S D 10.9; Retest sample Mean 46.8, S D 11.0.

Navigate back to Table 1..

### Figure 2. Long description

The figure consists of five panels arranged in two rows. The y-axis for all panels represents parameter recovery r, ranging from 0.00 to 1.00. Horizontal dashed lines at 0.4, 0.6, and 0.75 divide the plot into shaded regions representing poor, fair, good, and excellent recovery.

Top row from left to right:

* Bandit panel: Five red data points for decay minus, decay plus, invtemp, lr minus, and lr plus. All points are in the excellent recovery zone above 0.75, with lr minus being the highest.

* Effort panel: Three olive-green points for accept, effsens, and rewsens. Accept is near 0.95, while effsens and rewsens are around 0.85.

* Explore panel: Two green points for theta R U and theta V, both positioned exactly at 1.00 with no error bars.

Bottom row from left to right:

* Gamble panel: Four blue points for invtemp, loss, risk minus, and risk plus. Invtemp and loss are in the good recovery zone around 0.70, while risk minus and risk plus are in the excellent zone above 0.75.

* Rbias panel: Four purple points for init V, instsens, lr, and rewsens. Init V, instsens, and lr show excellent recovery above 0.90. Rewsens is the outlier, falling in the fair recovery zone at approximately 0.45 with a large error bar extending from 0.40 to 0.55.

Navigate back to Figure 2..

### Figure 3. Long description

A multi-panel figure with three horizontal forest plots arranged side-by-side.

Axes and Scale:

All three plots share a common Y-axis labeled Parameter and an X-axis labeled Test-retest reliability (r) ranging from -1.0 to 1.0. Vertical dashed lines at 0.4, 0.6, and 0.75 divide the plots into reliability zones: poor (dark gray background), fair (medium gray), good (light gray), and excellent (white).

Panel 1: I C C (C,1)

Panel 2: I C C (A,1)

Panel 3: Joint model

Data Trends from Top to Bottom:

* Bandit model (red): Five parameters including Inverse Temperature and L R variants. Most points cluster between 0.4 and 0.6 (fair reliability).

* Effort model (olive): Three parameters including Effort sensitivity, Reward sensitivity, and Intercept. Points cluster between 0.5 and 0.7 (fair to good).

* Explore model (teal): Relative uncertainty and Value difference. Points are near 0.5 (fair).

* Gambling model (blue): Four parameters including Loss Aversion and Risk Aversion variants. Reliability ranges from 0.4 to 0.7 (fair to good).

* Reward bias model (magenta): Four parameters including Reward sensitivity, Instruction sensitivity, Initial value, and Learning rate. These show the highest variance, with points ranging from 0.0 (poor) to 0.8 (excellent).

The Joint model panel generally shows higher reliability estimates and narrower confidence intervals compared to the I C C panels, particularly for the Gambling and Reward bias models.

Navigate back to Figure 3..

### Figure 4. Long description

A multi-panel figure labeled A through E.

Panel A: A 3 by 3 correlation matrix for learning rate constructs. The diagonal is solid red. Off-diagonal cells show moderate positive correlations between B lr plus and B lr minus, and B lr minus and Rb lr.

Panel B: A 5 by 5 correlation matrix for inverse temperature constructs. It shows weak to moderate correlations between parameters from Ex, Rb, Ef, G, and B tasks.

Panel C: A posterior distribution plot. The x-axis ranges from 0.1 to 0.5. A teal curve peaks at approximately 0.35. A vertical line marks the median at 0.35, with a shaded teal region representing the 95 percent mass interval between approximately 0.31 and 0.38.

Panel D: A large 18 by 18 exploratory correlation matrix. The diagonal is solid red. A color scale on the right indicates red for positive 1.0, white for 0.0, and blue for negative 1.0. Most off-diagonal cells are white, indicating non-significant correlations, with a few light red or blue squares visible in the bottom-left corner involving B task parameters.

Panel E: Four bar charts showing factor loadings for five tasks: B (salmon), Ef (olive), Ex (green), G (blue), and Rb (pink).

- Top chart: Bandit parameters factor shows high positive loadings for B lr plus and B decay minus, and negative loadings for B decay plus and B lr minus.

- Second chart: Sensitivity factor shows positive loadings for Ef rewsens, G risk plus, G risk minus, and G loss.

- Third chart: Uncertainty or risk factor shows a strong negative loading for G invtemp and positive loadings for Ex thetaV and Ex thetaRU.

- Bottom chart: Inverse temperature factor shows positive loadings for B invtemp and Ef acc.

Navigate back to Figure 4..

### Figure 5. Long description

The figure consists of seven panels labeled A through G.

Panels A, B, and C are dot-and-whisker plots showing correlation coefficients on the y-axis ranging from minus 0.2 to 0.1.

* Panel A compares Bandit negative learning rate and Effort reward sensitivity against Anxiety or depression (dark blue) and Zung Score (light blue). Effort reward sensitivity shows a significant negative correlation marked with an asterisk.

* Panel B compares Effort effort sensitivity and Reward bias reward sensitivity against Motivation or interest (dark green) and T E P S Score (light green). Reward bias shows a significant negative correlation.

* Panel C compares Effort reward sensitivity against Motivation or interest and A E S Score (yellow-green).

Panels D and E are linear regression line graphs with shaded 95 percent confidence intervals.

* Panel D shows a negative linear relationship between Anxiety or depression on the x-axis and Effort reward sensitivity on the y-axis.

* Panel E shows a negative linear relationship between Motivation or interest on the x-axis and Reward bias reward sensitivity on the y-axis.

Panels F and G are posterior distribution density plots from M C M C draws.

* Panel F shows a blue distribution curve with a central median line and a shaded 95 percent mass interval centered around 0.1.

* Panel G shows a green distribution curve with a central median line and a shaded 95 percent mass interval centered around 0.

Navigate back to Figure 5..

### Figure 6. Long description

A two-panel figure displaying correlation matrices. To the right of the panels is a vertical color scale labeled Corr, ranging from negative 1.0 in blue to 0.0 in white, and positive 1.0 in red.

Panel A: Correlations between model parameters and four exploratory factors. The Y-axis lists 18 parameters grouped by task: Reward Bias (R b), Explore (Ex), Gamble (G), Effort (Ef), and Bandit (B). The X-axis lists four factors: Anxiety/depression, Motivation/interest, Fatigue, and Compulsivity. Most cells are white, indicating non-significance. Notable colored cells include:

* G: invtemp shows a light red positive correlation with Motivation/interest and a light blue negative correlation with Compulsivity.

* Ef: effsens shows a light blue negative correlation with Fatigue.

* B: invtemp and B: l r minus show light red positive correlations with Compulsivity.

Panel B: Correlations between the same 18 model parameters and seven questionnaire scores: F S S Score, A E S Score, Zung Score, S T A I S Score, S T A I T Score, O C I R Score, and T E P S Score. Significant correlations are sparse, including:

* G: invtemp shows light blue negative correlation with O C I R Score and light red positive correlation with T E P S Score.

* Ef: rewsens shows light blue negative correlation with S T A I S and S T A I T Scores.

* B: l r minus shows a light red positive correlation with O C I R Score.

Navigate back to Figure 6..
