# Supplementary material for: The recoverability, reliability, and generalizability of reward processing parameters and relation to mental health symptoms
Source: Psychol Med. 2026 Jul 28;56:e238. doi: 10.1017/S0033291726105340 (PMC13439255; doi:10.1017/S0033291726105340)
Supplement: Supplementary file 1 [file S0033291726105340sup001.pdf]

Supplementary material

Supplementary methods

Additional questionnaires

Along with the main questionnaires listed in the text, participants also completed the International Physical Activity Questionnaire (IPAQ, 7 day/short version (Craig et al., 2003)) to examine exercise levels, a relevant confound when examining effort and anhedonia, and a COVID-19 impact questionnaire as some data were collected during 2020 (Anet, Agatha et al., 2020). Catch questions that required participants to set a slider bar at a specific location were integrated into the questionnaires as attention checks.

STAI versions

There are several different versions of the Spielberger State-Trait Inventory, and we used a revised version (Spielberger & Diaz-Guerrero, 1983). It is worth noting that this has several items which are different from the more commonly used versions. The items we used are listed below:

STAI-T

I feel pleasant
I feel nervous and restless
I feel satisfied with myself
I wish I could be as happy as others seem to be
I feel like a failure
I feel rested
I am 'calm, cool and collected'
I feel that difficulties are piling up so that I cannot overcome them
I worry too much over something that really doesn't matter
I am happy
I have disturbing thoughts
I lack self-confidence
I feel secure
I make decisions easily
I feel inadequate
I am content
Some unimportant thought runs through my mind and bothers me
I take disappointments so keenly that I can't put them out of my mind
I am a steady person
I get in a state of tension or turmoil as I think over my recent concerns and interests

STAI-S

I feel calm
I feel secure

I am tense
I am regretful
I feel at ease
I feel upset
I am presently worrying about possible misfortunes
I feel rested
I feel anxious
I feel comfortable
I feel self confident
I feel nervous
I am jittery
I feel "high strung"
I am relaxed
I feel content
I am worried
I feel over-excited and rattled
I feel joyful
I feel pleasant

Power calculation

An *a priori* power calculation using G*Power (Faul et al., 2007) indicated that to enable detection of $r = 0.15$ with $\alpha = 0.05$ (two tailed) and power = 0.9, 459 participants were required. To account for data exclusion (a typical exclusion rate for online research is ~25%(Peer et al., 2022; Zorowitz, Solis, et al., 2023)), we aimed to recruit 600 participants. For the test-retest analyses, 115 participants provides 95% power to observe r=0.3 at $\alpha = 0.05$ (one-tailed); one-tailed tests are appropriate here as negative intraclass correlation coefficients (ICCs) cannot be interpreted.

Inference

Inference was performed in a number of ways depending on the assumption being tested.

All reported exploratory factor analyses were computed using the ‘minimum residual’ method, with oblique rotation (oblimin) using the ‘Psych’ package in R. The number of factors was determined based on a scree test (Cattell, 1966), and factor scores were calculated using Thurstone’s method.

Where possible, we complement a conventional frequentist analysis with one in which the estimate of the effect is embedded within the model and estimated as part of the hierarchical Bayesian MCMC process. This latter procedure should represent a realistic ‘upper-bound’ estimate of the numeric values associated with many of the assumptions we test: by estimating the relevant quantity for each sample, the additional noise introduced by taking a mean of a parameter and performing a statistical test post-hoc is removed (Haines et al., 2025; Mkrtchian et al., 2023; Moutoussis et al., 2018; Pike et al., 2024).

Where we complement our analyses with qualitative interpretations of the levels of reliability, we use the conventions proposed by Cicchetti: 'poor' is below 0.40, 'fair' is 0.40 to 0.60, 'good' is 0.60 to 0.75, and above 0.75 is 'excellent' (Cicchetti, 1994). Note that these are just suggestions to aid interpretation, rather than meaningful 'categories' of reliability or empirically-derived thresholds.

Supplementary results

Model-agnostic test-retest reliability and practice effects

We also assessed the test-retest reliability of model-agnostic variables from the five tasks. These were variables estimated for various mixed logistic regression models, incorporating a similar trial-by-trial analysis to our computational models. These were estimated separately for each session, and also jointly, with random intercepts and slopes with session as a grouping variable, allowing for the variance to be extracted and an ICC(1) to be calculated - mirroring the approach taken with model parameters. The ICCs in Supplementary Table 1 are calculated on the basis of the predictions from these logistic regressions for different conditions.

For the bandit task, the logistic regression for the full joint model was: $stay \sim previousOutcome \ + \ (1 \ + \ previousOutcome|id/session)$, with relevant conditions being the probability of staying given a win, loss, both a win and a loss, or neither.

For the effort task, the logistic regression was: $accept \sim reward * effort \ + \ (1 + reward * effort|id/session)$. These conditions were treated continuously, so the calculation is just of the probability of accepting.

And for the explore task: $choseRisky \sim previousReward \ + \ (1 \ + \ previousReward|id/session)$: here the probability of choosing the risky option is predicted for each individual and each session.

And the gambling task: $gamble \sim condition \ + \ (1 + condition|id/session)$, with the conditions being the probability of gambling on loss-only, gain-only and mixed trials.

Finally, the equation for the reward bias task was: $choseRich \sim coherence \ + \ (1 + coherence|id/session)$, with the conditions being the different levels of coherence of the random dot kinematograms.

Supplementary Table 1: Test-retest reliability of task parameters. ICC(1) refers to a one-way intraclass correlation coefficient, ICC(A,1) to a two-way absolute ICC, and Pearson's *r* to correlation estimates. Joint model here refers to the ICC(1) estimated using a single mixed-effects logistic regression with random intercepts and slopes, accounting for data for both sessions by including session as a second-level grouping factor with subjects nested within session (Brown et al., 2020; Mkrtchian et al., 2023; Waltmann et al., 2022).

Measure	ICC(A,1)	ICC(1)	Pearson's *r*	Joint model
**Bandit task: p(stay) logistic regression**				
Win	0.565 (0.37–0.70)	0.549 (0.41–0.67)	0.608 (0.48–0.71)	0.579
Loss	0.533 (0.38–0.65)	0.525 (0.38–0.65)	0.560 (0.42–0.67)	0.586
Neither	0.621 (0.49–0.79)	0.617 (0.49–0.72)	0.638 (0.51–0.74)	0.639
Both	0.551 (0.36-0.69)	0.534 (0.39-0.65)	0.600 (0.47-0.71	0.566
**Effort task: p(accept) logistic regression**				
P(accept)	0.723 (0.69-0.75)	0.721 (0.70-0.74)	0.744 (0.72-0.76)	0.730
**Explore task: p(chose risky) logistic regression**				
P(Chose Risky)	0.261 (0.16-0.36)	0.255 (0.15-0.35)	0.266 (0.16-0.36)	0.427
**Gamble task: p(gamble) logistic regression**				
Loss-only trials	0.542 (0.40–0.66)	0.536 (0.39–0.65)	0.615 (0.49–0.72)	0.917
Mixed trials	0.684 (0.57–0.77)	0.682 (0.57–0.77)	0.69 (0.58–0.78)	0.887
Gain-only trials	0.598 (0.47–0.70)	0.598 (0.47–0.70)	0.600 (0.47–0.71)	0.869
**Reward bias task: p(chose rich) logistic regression**				
0.5 coherence	0.334 (0.16-0.49)	0.333 (0.16-0.49)	0.338 (0.17-0.49)	0.394
0.6 coherence	0.363 (0.19-0.51)	0.365 (0.20-0.51)	0.367 (0.20–0.52)	0.439

Measure	ICC(A,1)	ICC(1)	Pearson's *r*	Joint model
0.8 coherence	0.411 (0.25-0.55)	0.408 (0.24-0.55)	0.42 (0.26-0.56)	0.593

We also estimated practice effects using paired t-tests. Within the bandit task, there was evidence for a practice effect on total score (M_{diff}=4.22, t_{114}=2.25, p=0.027). There was also evidence of a decrease in the number of offers accepted in the effort task between times 1 and 2 (M_{diff}=-2.97, t_{114}=-2.58, p=0.011). There was also an increase in rewards obtained in the explore task (M_{diff}=277.15, t_{114}=4.85, p<0.001). There was, however, no evidence of practice effects in the number of gambles in the gambling task (M_{diff}=-0.59, t_{114}=-1.20, p=0.232), or the rewards in the reward bias task (M_{diff}=0.35, t_{114}=0.99, p=0.323).

Model comparison

The best fitting model, as shown by integrated BIC, is shown in Supplementary Figure 1 below. Note that for the 'effort' task, model 4 was the best fitting but also had an unsatisfactory traceplot, indicating poor chain mixing, so model 2 was preferred. See below for these traceplots.

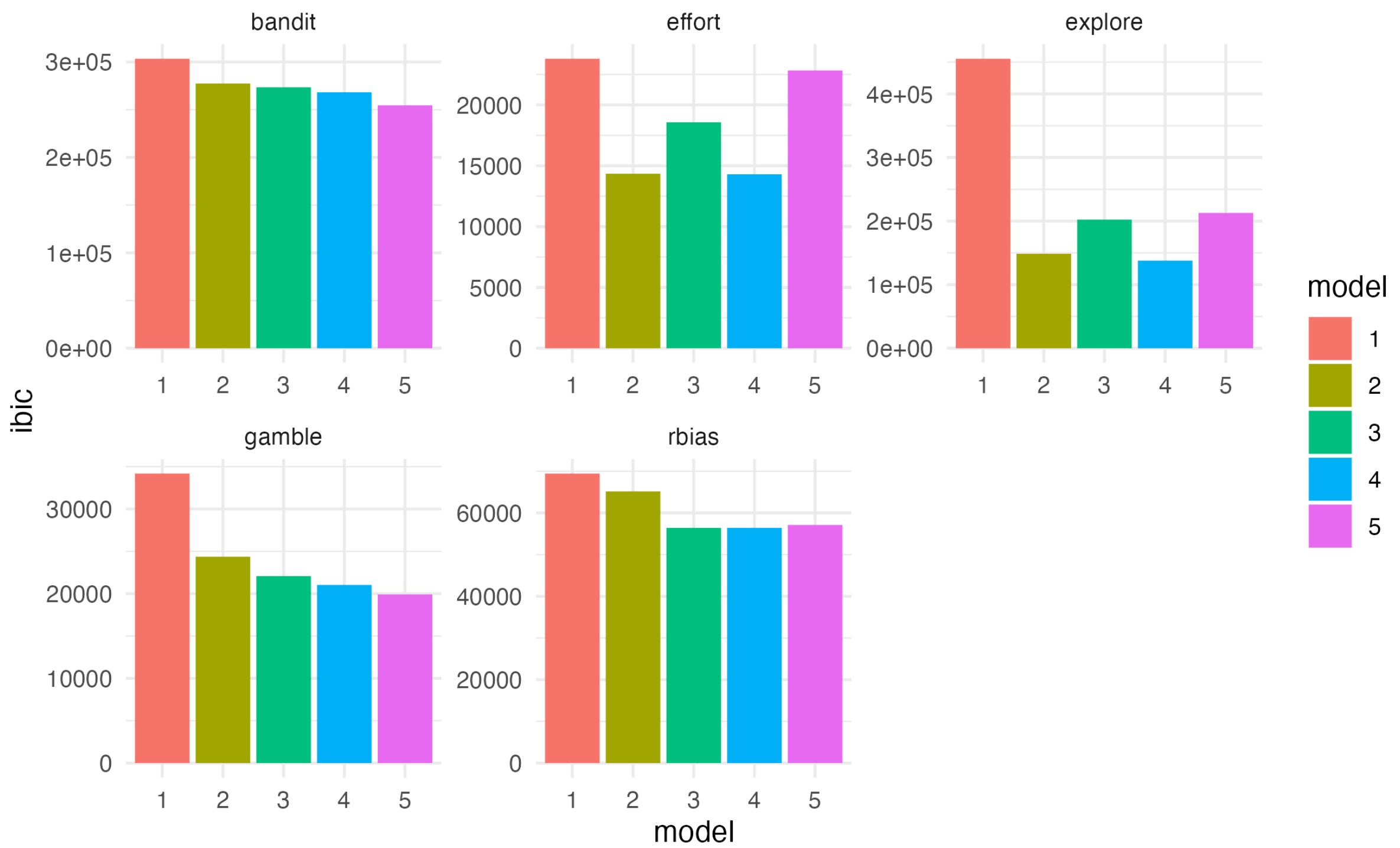

Supplementary Figure 1: Bar plots of integrated BIC for each of the five models tested on the five tasks. Lower integrated BIC represents a better fit to the data.

The below shows the log posterior traceplot for model 4 of the effort task, indicating poor chain mixing. Model 2 (on the left) shows much better mixing, and had a very similar iBIC score (see Supplementary Figure 1 above), so this model was selected.

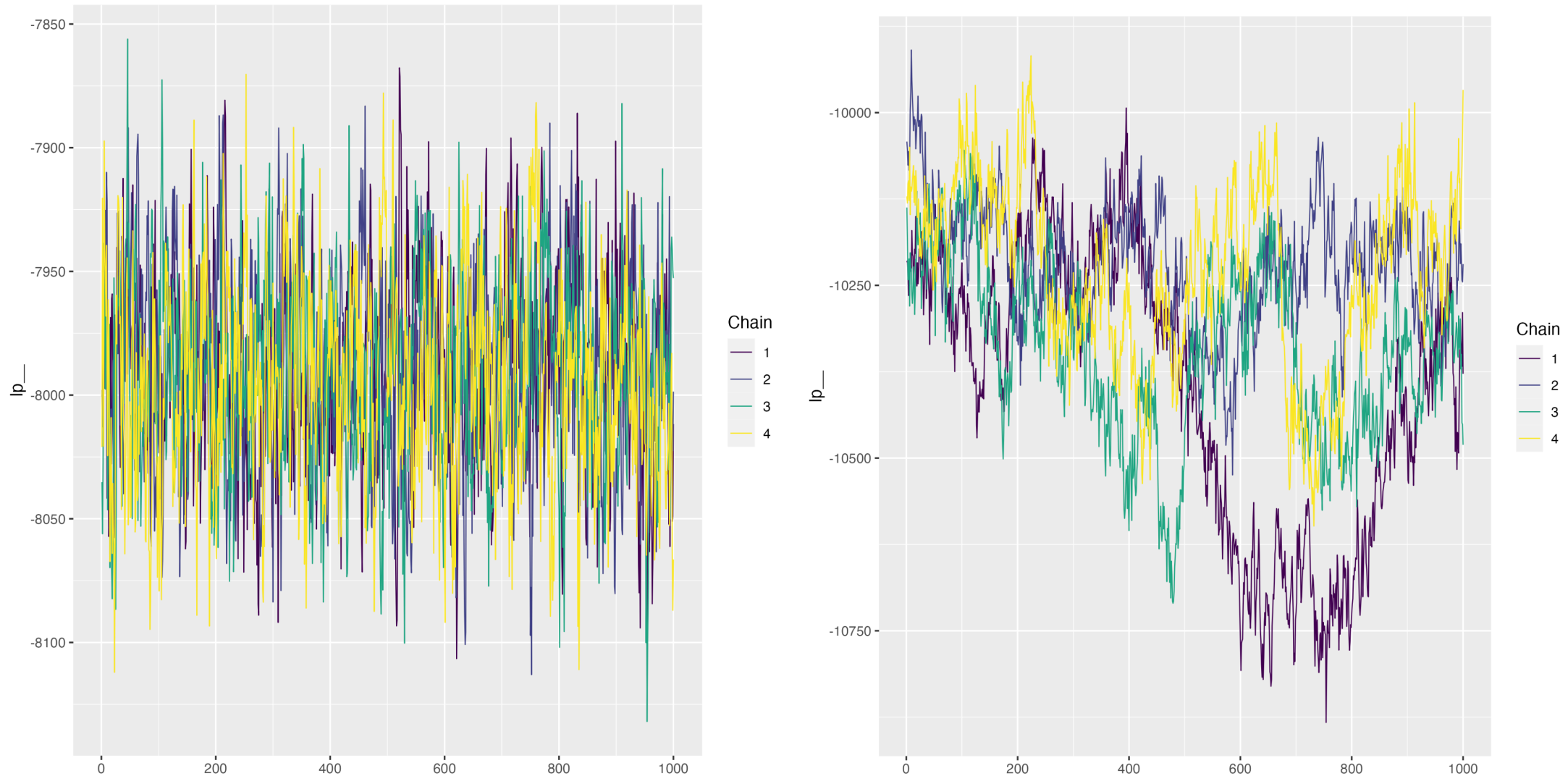

Supplementary Figure 2: Trace plots of the log posterior for model 2 and model 4 of the effort task respectively. Model 2 chains are non-overlapping, indicating poor mixing.

Note that in the reward bias task, model 5 performs better than model 3 for test-retest. Users of this task in the future may wish to consider test-retest performance as a metric for model selection.

Parameters are stable over time

Supplementary Table 2: Test-retest reliability of task parameters. ICC(C,1) refers to consistency intraclass correlation coefficient, ICC(A,1) to absolute ICC, and 'joint model' refers to the correlation coefficient estimated by embedding the correlation matrix within the model.

**Parameters**	**ICC(C,1)**	**ICC(A,1)**	**Joint model**
**Bandit task**			
Inverse temperature	0.36 (0.20 - 0.51)	0.36 (0.20 - 0.51)	0.66 (0.52 - 0.78)
LR unchosen -EV	0.43 (0.26 - 0.56)	0.43 (0.27 - 0.57)	0.67 (0.51 - 0.80)
LR chosen -EV	0.43 (0.27 - 0.57)	0.43 (0.27 - 0.57)	0.54 (0.38 - 0.68)
LR unchosen +EV	0.43 (0.26 - 0.56)	0.43 (0.26 - 0.56)	0.80 (0.67 - 0.89)
LR chosen +EV	0.42 (0.25 - 0.56)	0.43 (0.26 - 0.57)	0.57 (0.43 - 0.70)
**Effort task**			

Parameters	ICC(C,1)	ICC(A,1)	Joint model
Effort sensitivity	0.69 (0.58 - 0.77)	0.69 (0.58 - 0.77)	0.88 (0.77 - 0.96)
Reward sensitivity	0.47 (0.31 - 0.6)	0.48 (0.32 - 0.61)	0.77 (0.59 - 0.90)
Acceptance bias	0.68 (0.57 - 0.77)	0.69 (0.54 - 0.79)	0.85 (0.78 - 0.91)
**Explore task**			
Relative uncertainty	0.45 (0.29 - 0.58)	0.48 (0.25 - 0.64)	0.62 (0.51 - 0.72)
Total uncertainty	0.38 (0.21 - 0.52)	0.44 (0.07 - 0.67)	0.64 (0.53 - 0.74)
**Gamble task**			
Inverse temperature	0.41 (0.25 - 0.55)	0.42 (0.25 - 0.56)	0.73 (0.55 - 0.88)
Loss aversion	0.64 (0.51 - 0.73)	0.64 (0.51 - 0.73)	0.95 (0.88 - 1.00)
Risk aversion loss	0.54 (0.40 - 0.66)	0.55 (0.41 - 0.66)	0.83 (0.73 - 0.91)
Risk aversion gain	0.63 (0.50 - 0.73)	0.63 (0.50 - 0.73)	0.88 (0.8 - 0.94)
**Reward bias task**			
Reward sensitivity	0.00 (-0.18 - 0.18)	0.00 (-0.18 - 0.18)	-0.02 (-0.91 - 0.90)
Instruction sensitivity	0.58 (0.45 - 0.69)	0.59 (0.43 - 0.71)	0.90 (0.79 - 0.97)
Initial value	0.09 (-0.10 - 0.26)	0.11 (-0.07 - 0.28)	0.09 (-0.07 - 0.26)
Learning rate	0.04 (-0.14 - 0.22)	0.05 (-0.13 - 0.23)	0.07 (-0.86 - 0.91)

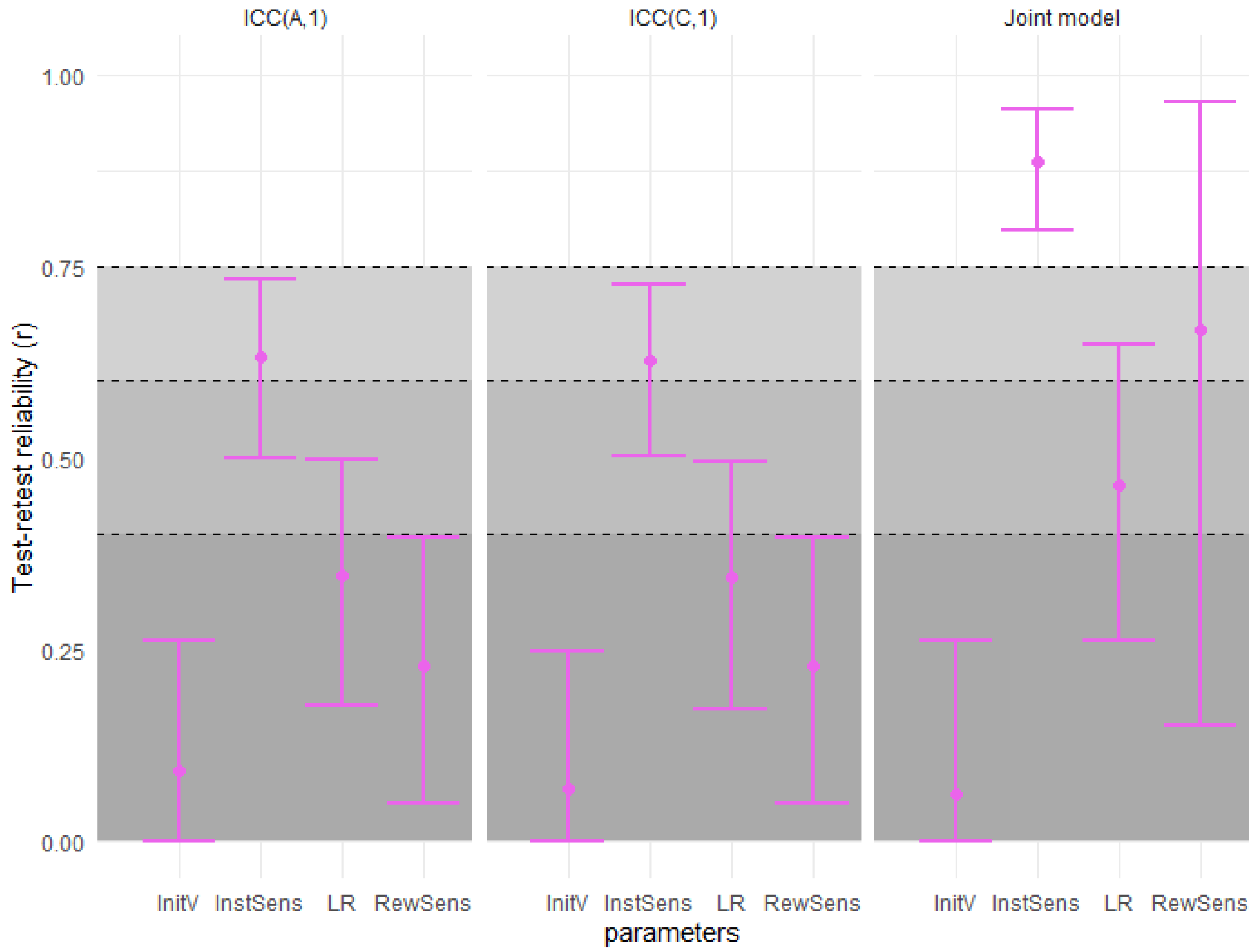

Supplementary Figure 3: Test-retest reliability for model 5 of the reward bias task is better than that reported in the main manuscript for model 3 (see main Figure 4 and Supplementary Table 2 above).

Conceptually related parameters do not generalise across task contexts

Supplementary Table 3: Relationship between parameters from different tasks, and the construct the parameter purports to measure. The latter two columns show that the inference does not change when using a linear regression model, controlling for z-scored age and gender. A * in the p-value column denotes statistical significance at a conventional alpha<0.05.

Construct	Task and parameter 1	Task and parameter 2	Correlation estimate	P value	Estimate controlling for age and gender	P value controlling for age and gender
Learning rate	Bandit: positive learning rate	Reward bias: learning rate	0.068	0.129	0.01	0.063
Learning rate	Bandit: negative learning rate	Reward bias: learning rate	0.009	0.845	0	0.952

Construct	Task and parameter 1	Task and parameter 2	Correlation estimate	P value	Estimate controlling for age and gender	P value controlling for age and gender
Inverse temperature	Bandit: inverse temperature	Effort: reward sensitivity	0.049	0.251	0.258	0.237
Inverse temperature	Bandit: inverse temperature	Explore: value difference	0.175	<0.001*	0.456	<0.001*
Inverse temperature	Bandit: inverse temperature	Gamble: inverse temperature	-0.077	0.071	-0.439	0.131
Inverse temperature	Bandit: inverse temperature	Reward bias: reward sensitivity	-0.080	0.075	-1.912	0.063
Inverse temperature	Effort: reward sensitivity	Explore: value difference	-0.105	0.014*	-0.054	0.020*
Inverse temperature	Effort: reward sensitivity	Gamble: inverse temperature	0.140	0.001*	0.177	0.002*
Inverse temperature	Effort: reward sensitivity	Reward bias: reward sensitivity	0.108	0.016*	0.501	0.015*
Inverse temperature	Explore: value difference	Gamble: inverse temperature	-0.227	<0.001*	-0.525	<0.001*
Inverse temperature	Explore: value difference	Reward bias: reward sensitivity	-0.118	0.008*	-1.063	0.005*
Inverse temperature	Gamble: inverse temperature	Reward bias: reward sensitivity	0.157	<0.001*	0.611	<0.001*

Exploratory factor analysis

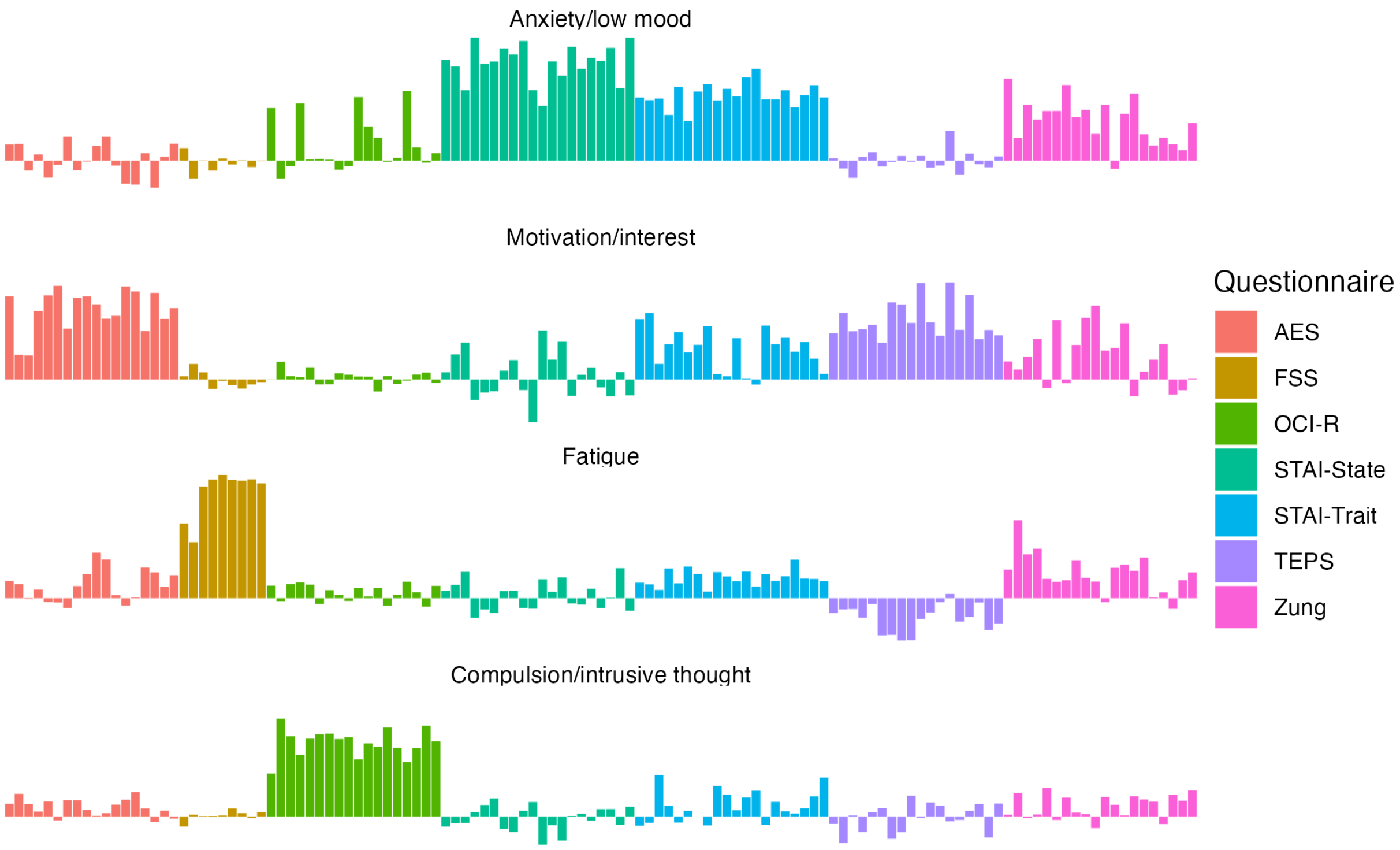

Supplementary Figure 4: Results of an exploratory factor analysis on the questionnaire items used in this study. A four-factor solution produced factors we named 'anxiety/low mood', 'motivation/interest', 'fatigue', and 'compulsion/intrusive thought'. The questionnaire the items are drawn from is shown on the legend. Bar height represents the loading of each item on the relevant factor.

Parameters relate weakly to symptoms

Supplementary Table 4: Relationship between parameters and mental health constructs. The latter two columns show that the inference does not change when using a linear regression model, controlling for z-scored age and gender. A * in the p-value column denotes statistical significance at a conventional alpha<0.05.

**Construct**	**Mental health measure**	**Task and parameter**	**Correlation estimate**	**P value**	**Estimate controlling for age and gender**	**P value controlling for age and gender**
Depression	Anxiety/ depression factor	Bandit: negative learning rate	0.073	0.088	0.021	0.156
Depression	Zung Score	Bandit: negative learning rate	0.071	0.099	0.002	0.169

Construct	Mental health measure	Task and parameter	Correlation estimate	P value	Estimate controlling for age and gender	P value controlling for age and gender
Depression	Anxiety/ depression factor	Effort: reward sensitivity	-0.098	0.021*	-0.091	0.030*
Depression	Zung Score	Effort: reward sensitivity	-0.076	0.077	-0.007	0.083
Anhedonia	Motivation/ interest factor	Effort: effort sensitivity	-0.040	0.351	-0.001	0.388
Anhedonia	TEPS Score	Effort: effort sensitivity	0.020	0.645	0.000	0.921
Anhedonia	Motivation/ interest factor	Reward bias: reward sensitivity	-0.101	0.023*	-0.458	0.021*
Anhedonia	TEPS Score	Reward bias: reward sensitivity	-0.063	0.156	-0.022	0.195
Apathy	Motivation/ interest factor	Effort: reward sensitivity	-0.016	0.715	-0.027	0.520
Apathy	AES Score	Effort: reward sensitivity	-0.037	0.385	-0.005	0.279
