# Supplementary material for: The recoverability, reliability, and generalizability of reward processing parameters and relation to mental health symptoms
Source: Psychol Med. 2026 Jul 28;56:e238. doi: 10.1017/S0033291726105340 (PMC13439255; doi:10.1017/S0033291726105340)
Supplement: Supplementary file 2 [file S0033291726105340sup002.pdf]

MSc Project Model Equations

Bandit task

Note, tau is 1/inverse.temperature for all of these. High tau indicates randomness, low inverse temperature indicates randomness.

Unless otherwise stated, all values are initialized at 0.

Model 1

Null model

Parameters: inverse temperature

$$ChoiceProbability_{option,t} = \frac{exp(InverseTemperature*Value_{option,t})}{\sum_{option=1}^{numberoptions} exp(InverseTemperature*Value_{option,t})}$$

Model 2

RL Model

Parameters: learning rate, inverse temperature

$$ChoiceProbability_{option,t} = \frac{exp(InverseTemperature*Value_{option,t})}{\sum_{option=1}^{numberoptions} exp(InverseTemperature*Value_{option,t})}$$

$$outcome_{option,t} = reward_{option,t} - loss_{option,t}$$

$$PredictionError_{t} = outcome_{option,t} - Value_{option,t}$$

$$Value_{option,t+1} = Value_{option,t} + LearningRate * PredictionError_{t}$$

Model 3

RL Model

Parameters: learning rate rewards, learning rate losses, inverse temperature

$$ChoiceProbability_{option,t} = \frac{exp(InverseTemperature*Value_{option,t})}{\sum_{option=1}^{numberoptions} exp(InverseTemperature*Value_{option,t})}$$

$$RewardPredictionError_{t} = reward_{option,t} - RewardValue_{option,t}$$

$$LossPredictionError_{t} = -\ loss_{option,t} - LossValue_{option,t}$$

$$RewardValue_{option,t+1} = RewardValue_{option,t} + RewardLearningRate * RewardPredictionError_{t}$$

$$LossValue_{option,t+1} = LossValue_{option,t} + LossLearningRate * LossPredictionError_{t}$$

$$Value_{option,t} = RewardValue_{option,t} + LossValue_{option,t}$$

Model 4

RL Model

Parameters: learning rate rewards, learning rate punishments, reward sensitivity, punishment sensitivity, lapse

$$ChoiceProbability_{option,t} = \frac{\exp exp\left(Value_{option,t}\right)}{\sum_{option=1}^{numberoptions} \exp exp\left(Value_{option,t}\right)} * (1 - lapse) + \frac{lapse}{4}$$

$$RewardPredictionError_{t} = RewardSensitivity * reward_{option,t} - RewardValue_{option,t}$$

$$LossPredictionError_{t} = -(LossSensitivity * loss_{option,t}) - LossValue_{option,t}$$

$$RewardValue_{option,t+1} = RewardValue_{option,t} + RewardLearningRate * RewardPredictionError_{t}$$

$$LossValue_{option,t+1} = LossValue_{option,t} + LossLearningRate * LossPredictionError_{t}$$

For unchosen options, there is fictive updating:

$$FictiveRewardPredictionError_{t} =- RewardValue_{option,t}$$

$$FictiveLossPredictionError_{t} =- LossValue_{option,t}$$

$$RewardValue_{option,t+1} = RewardValue_{option,t} + RewardLearningRate * FictiveRewardPredictionError_{t}$$

$$LossValue_{option,t+1} = LossValue_{option,t} + LossLearningRate * FictiveLossPredictionError_{t}$$

And finally

$$Value_{option,t} = RewardValue_{option,t} + LossValue_{option,t}$$

Model 5

RL Model

Parameters: positive and negative learning rates for chosen and unchosen options, inverse temperature

$$ChoiceProbability_{option,t} = \frac{exp(InverseTemperature*Value_{option,t})}{\sum_{option=1}^{numberoptions} exp(InverseTemperature*Value_{option,t})}$$

Where 'option' refers to the stimulus participants selected, updates are performed as follows:

$$RewardPredictionError_{option,t} = reward_{option,t} - RewardValue_{option,t}$$

$$LossPredictionError_{option,t} = - loss_{option,t} - LossValue_{option,t}$$

If reward prediction error>0

$$RewardValue_{option,t+1} = RewardValue_{option,t} + PositiveLearningRateChosen * RewardPredictionError_{option,t}$$

Otherwise

$$RewardValue_{option,t+1} = RewardValue_{option,t} + NegativeLearningRateChosen * RewardPredictionError_{option,t}$$

Similarly, for loss prediction errors:

If loss prediction error >0

$$LossValue_{option,t+1} = LossValue_{option,t} + PositiveLearningRateChosen * LossPredictionError_{option,t}$$

Otherwise

$$LossValue_{option,t+1} = LossValue_{option,t} + NegativeLearningRateChosen * LossPredictionError_{option,t}$$

A similar logic is used to update all of the other stimuli which are not chosen (here denoted by 'unchosen' rather than 'option' for clarity):

$$RewardPredictionError_{unchosen,t} = 0 - RewardValue_{unchosen,t}$$

$$LossPredictionError_{unchosen,t} = 0 - LossValue_{unchosen,t}$$

If reward prediction error for the unchosen option >0

$$RewardValue_{unchosen,t+1} = RewardValue_{unchosen,t} + PositiveLearningRateUnchosen * RewardPredictionErr$$

Otherwise

$$RewardValue_{unchosen,t+1} = RewardValue_{unchosen,t} + NegativeLearningRateUnchosen * RewardPredictionEr$$

Similarly, for loss prediction errors:

If loss prediction error for the unchosen stimulus >0

$$LossValue_{unchosen,t+1} = LossValue_{unchosen,t} + PositiveLearningRateUnchosen * LossPredictionError_{unchosen,t}$$

Otherwise

$$LossValue_{unchosen,t+1} = LossValue_{unchosen,t} + NegativeLearningRateUnchosen * LossPredictionError_{unchosen}$$

EFFORT TASK

Model 1

Null model

Parameters: intercept

$$ProbabilityAccept_t = \frac{\exp exp\,(intercept)}{1+\exp exp\,(intercept)}$$

Model 2

Logistic model

Parameters: intercept, linear reward, linear effort

$$ProbabilityAccept_t = \frac{\mathrm{exp}exp\left(intercept+reward*RewardLevel_t+effort*EffortLevel_t\right)}{1+\mathrm{exp}exp\left(intercept+reward*RewardLevel_t+effort*EffortLevel_t\right)}$$

Model 3

Logistic model

Parameters: intercept, linear reward, linear effort, quadratic effort (effort2)

$$ProbabilityAccept_t = \frac{\mathrm{exp}exp\left(intercept+reward*RewardLevel_t+effort*EffortLevel_t+effort2*EffortLevel_t^2\right)}{1+\mathrm{exp}exp\left(intercept+reward*RewardLevel_t+effort*EffortLevel_t+effort2*EffortLevel_t^2\right)}$$

Model 4

Robust logistic model

Parameters: intercept, linear reward, linear effort, lapse

$$ProbabilityAccept_t = \frac{lapse}{2} + (1 - lapse) * \frac{\mathrm{exp}exp\left(intercept+reward*RewardLevel_t+effort*EffortLevel_t\right)}{1+\mathrm{exp}exp\left(intercept+reward*RewardLevel_t+effort*EffortLevel_t\right)}$$

Model 5

Robust logistic model

Parameters: intercept, linear reward, linear effort, quadratic effort, lapse

$$ProbabilityAccept_t = \frac{lapse}{2} + (1 - lapse) * \frac{\mathrm{exp}exp\left(intercept+reward*RewardLevel_t+effort*EffortLevel_t+effort2*EffortLevel\right.}{1+\mathrm{exp}exp\left(intercept+reward*RewardLevel_t+effort*EffortLevel_t+effort2*EffortLev\right.}$$

Gambling Task

Model 1

Null Model

Parameters: inverse temperature

$$ProbabilityGamble_t = \frac{exp(InverseTemperature*(ValueGamble-ValueSafe))}{1+exp(InverseTemperature*(ValueGamble-ValueSafe))}$$

Note that here, ValueGamble and ValueSafe are set to 0.5 and not updated, so in essence

$$ProbabilityGamble_t = \frac{exp(InverseTemperature*0)}{1+exp(InverseTemperature*0)}$$

And therefore

$$ProbabilityGamble_t = \frac{exp(0)}{1+exp(0)}$$

So the probability of gambling on each trial is 0.5

Model 2

Risk aversion model

Parameters: risk aversion, inverse temperature

Note that Certain is the value of the certain option, GambleReward is the amount participants could win if they select the 'win' option, and GambleLoss is the amount they could lose if they select the loss option. All losses should be written as negative numbers. All probabilities of gambles in this version of the task are 0.5.

$$ProbabilityGamble_t = \frac{exp(InverseTemperature*(ValueGamble_t - ValueSafe_t))}{1+exp(InverseTemperature*(ValueGamble_t - ValueSafe_t))}$$

Where

$$ValueSafe_t = Certain^{RiskAversion}$$

$$ValueGamble_t = 0.5 * GambleReward^{RiskAversion} + 0.5 * GambleLoss^{RiskAversion}$$

Model 3

Loss aversion model

Parameters: loss aversion, inverse temperature

$$ProbabilityGamble_t = \frac{exp(InverseTemperature*(ValueGamble_t - ValueSafe_t))}{1+exp(InverseTemperature*(ValueGamble_t - ValueSafe_t))}$$

Where on loss-only trials

$$ValueSafe_t = LossAversion * Certain^1$$

And otherwise

$$ValueSafe_t = Certain^1$$

And

$$ValueGamble_t = 0.5 * (GambleReward^1 + LossAversion * GambleLoss^1)$$

Model 4

Risk and loss aversion model

Parameters: risk aversion, loss aversion, inverse temperature

$$ProbabilityGamble_t = \frac{exp(InverseTemperature*(ValueGamble_t - ValueSafe_t))}{1+exp(InverseTemperature*(ValueGamble_t - ValueSafe_t))}$$

Where on loss-only trials

$$ValueSafe_t = LossAversion * Certain^{RiskAversion}$$

And otherwise

$$ValueSafe_t = Certain^{RiskAversion}$$

And

$$ValueGamble_t = 0.5 * (GambleReward^{RiskAversion} + LossAversion * GambleLoss^{RiskAversion})$$

Model 5

Separate risk and loss aversion model

Parameters: risk aversion gain trials, risk aversion loss trials, loss aversion, inverse temperature

$$ProbabilityGamble_t = \frac{exp(InverseTemperature*(ValueGamble_t - ValueSafe_t))}{1+exp(InverseTemperature*(ValueGamble_t - ValueSafe_t))}$$

For loss-only trials

$$ValueSafe_t = LossAversion * Certain^{LossRiskAversion}$$

For gain-only trials

$$ValueSafe_t = Certain^{GainRiskAversion}$$

Note that for mixed trials the amount participants can win by choosing the safe option is 0, and as y*0^x is 0, $ValueSafe_t$ is also 0.

And

$$ValueGamble_t = 0.5 * (GambleReward^{GainRiskAversion} + LossAversion * GambleLoss^{LossRiskAversion})$$

Exploration Task

Note that these models all use various outputs from the Kalman Filter. These values are updated iteratively, on each trial, and there are no free parameters fit to each individual.

Here, each option's value is represented as a distribution, with a mean *m* and variance *v*. The aim of the Kalman filter is to track how the posterior estimates of these distributions are updated. Stimuli distributions are initialized with a mean of 0, and a variance of 100.

The Kalman gain is calculated as follows:

$$KalmanGain_t = \frac{s_{choice,t}}{s_{choice,t} + Q_{choice,t}}$$

$$Error_t = outcome_t - m_{choice,t}$$

$$m_{choice,t+1} = m_{choice,t} + KalmanGain_t * Error_t$$

$$s_{choice,t+1} = s_{choice,t} - KalmanGain_t * s_{choice,t}$$

$Q_{choice,t}$ is initialized at .0001 and 16 for the safe and risky option respectively.

The resulting key terms used in subsequent models are defined as follows:

$$ValueDiff_t = m_{2,t} - m_{1,t}$$

$$RelativeUncertainty_t = v_{2,t} - v_{1,t}$$

$$TotalUncertainty_t = \sqrt{v_{1,t} + v_{2,t}}$$

$$WeightedValueDiff_t = \frac{ValueDiff_t}{TotalUncertainty_t}$$

Model 1

Null Model

Parameters: intercept (aka inverse temperature here)

$$ChoiceProbability_{option,t} = \frac{exp(InverseTemperature*ValueDiff_{option,t})}{1+exp(InverseTemperature*ValueDiff_{option,t})}$$

Model 2

Value difference model

Parameters: ThetaV

This uses the 'Phi' function in Stan, which is the cumulative distribution function of the standard normal distribution.

$$ChoiceProbability_{option,t} = \phi(ThetaV * ValueDiff_{option,t})$$

Model 3

Weighted value difference model

Parameters: Theta_VTU

$$ChoiceProbability_{option,t} = \phi(ThetaVTU * Value/TotalUncertainty_{option,t})$$

Model 4

Value difference + sigma difference model

Parameters: Theta_V, Theta_RU

$$ChoiceProbability_{option,t} = \phi(\left(ThetaV * Value_{option,t}\right) + \left(ThetaRU * RelativeUncertainty_{option,t}\right))$$

Model 5

Hybrid model

Parameters: Theta_V, Theta_RU, Theta_VTU

$$ChoiceProbability_{option,t} = \phi(\left(ThetaV * Value_{option,t}\right) + \left(ThetaRU * RelativeUncertainty_{option,t}\right) + (ThetaVT$$

Reward Bias/Pizzagalli Task

Note that there is a separate learnt value for each option (left or right) for each congruence level (20%, 40%, 50%, 60%, 80% for the majority of these models, unless otherwise highlighted.

Model 1

Null Model

Parameters: inverse temperature

Note that this is the probability of choosing left or right *for a given congruence level*, so the sum on the denominator is only over the values of left and right.

$$ChoiceProbability_{option,t} = \frac{exp(InverseTemperature*Value_{option,congruence,t})}{\sum_{option=1}^{2} exp(InverseTemperature*Value_{option,congruence,t})}$$

Here, values are initialized at 0 and not updated.

Model 2

Win-stay lose-shift model

Parameters: inverse temperature

$$ChoiceProbability_{option,t} = \frac{exp(InverseTemperature*Value_{option,congruence,t})}{\sum_{option=1}^{2} exp(InverseTemperature*Value_{option,congruence,t})}$$

$$PredictionError_{t} = outcome_{option,t} - Value_{option,t}$$

$$Value_{congruence,option,t+1} = Value_{congruence,option,t} + PredictionError_{t}$$

No learning rate is included, so it is equivalent to a learning rate of 1 – after every win outcome, participants should 'stay', and otherwise, they should 'switch'.

Model 3

Stimulus-action model

Parameters: learning rate, reward sensitivity, instruction sensitivity, initial value

Here, *correct*$_t$ represents whether they were accurate in identifying the direction of motion of the dots. For 50% congruence, they were recorded as being 'accurate' 50% of the time, at random.

$$ChoiceProbability_{option,t} = \frac{exp(Value_{option,congruence,t}+InstructionSensitivity*correct_{t})}{\sum_{option=1}^{2} exp(Value_{option,congruence,t}+InstructionSensitivity*correct_{t})}$$

$$PredictionError_{t} = RewardSensitivity * outcome_{option,t} - Value_{option,t}$$

$$Value_{congruence,option,t+1} = Value_{congruence,option,t} + PredictionError_{t}$$

Initial values were estimated freely, such that on trial 0 for all congruence levels

$$Value_{congruence,left,1} = initialValue$$

And

$$Value_{congruence,right,1} = 1 - initialValue$$

Model 4

Stimulus-action model with separate sensitivities

Parameters: learning rate, reward sensitivity, punishment sensitivity, instruction sensitivity, initial value

$$ChoiceProbability_{option,t} = \frac{exp(Value_{option,congruence,t} + InstructionSensitivity * correct_t)}{\sum_{option=1}^{2} exp(Value_{option,congruence,t} + InstructionSensitivity * correct_t)}$$

If the outcome for making that choice was >0, then

$$PredictionError_t = RewardSensitivity * outcome_{option,t} - Value_{option,t}$$

Otherwise

$$PredictionError_t = PunishmentSensitivity * outcome_{option,t} - Value_{option,t}$$

$$Value_{congruence,option,t+1} = Value_{congruence,option,t} + PredictionError_t$$

Initial values were estimated freely, such that on trial 0 for all congruence levels

$$Value_{congruence,left,1} = initialValue$$

And

$$Value_{congruence,right,1} = 1 - initialValue$$

Model 5

Action-only model

Parameters: learning rate, reward sensitivity, instruction sensitivity, initial value

Key difference between this model and model 3 is there are no separate values for different congruence levels, so only one value for left and one value for right is learnt and updated.

$$ChoiceProbability_{option,t} = \frac{exp(Value_{option,t} + InstructionSensitivity * correct_t)}{\sum_{option=1}^{2} exp(Value_{option,t} + InstructionSensitivity * correct_t)}$$

$$PredictionError_t = RewardSensitivity * outcome_{option,t} - Value_{option,t}$$

$$Value_{option,t+1} = Value_{option,t} + PredictionError_t$$

Initial values were estimated freely, such that on trial 0 for all congruence levels

$$Value_{left,1} = initialValue$$

And

$$Value_{right,1} = 1 - initialValue$$
